# The *loop-tail* mouse model displays open and closed caudal neural tube defects

**DOI:** 10.1242/dmm.050175

**Published:** 2023-08-29

**Authors:** Beatriz Fernández-Santos, Marta Reyes-Corral, José Manuel Caro-Vega, Miguel Lao-Pérez, Claudia Vallejo-Grijalba, Cristina Mesa-Cruz, Francisco J. Morón, Patricia Ybot-González

**Affiliations:** Institute of Biomedicine of Seville (IBiS)/Virgen del Rocío University Hospital/CSIC/University of Seville, 41013 Seville, Spain

**Keywords:** Neural tube defects, *Loop-tail*, *Vangl2*, Spina bifida, Wnt-planar cell polarity pathway, Lipomyelomeningocele

## Abstract

Neural tube defects (NTDs) are the second most common cause of congenital malformations and are often studied in animal models. *Loop-tail* (*Lp*) mice carry a mutation in the *Vangl2* gene, a member of the Wnt-planar cell polarity pathway. In *Vangl2^+/Lp^* embryos, the mutation induces a failure in the completion of caudal neural tube closure, but only a small percentage of embryos develop open spina bifida. Here, we show that the majority of *Vangl2^+/Lp^* embryos developed caudal closed NTDs and presented cellular aggregates that may facilitate the sealing of these defects. The cellular aggregates expressed neural crest cell markers and, using these as a readout, we describe a systematic method to assess the severity of the neural tube dorsal fusion failure. We observed that this defect worsened in combination with other NTD mutants, *Daam1* and *Grhl3*. Besides, we found that in *Vangl2^+/Lp^* embryos, these NTDs were resistant to maternal folic acid and inositol supplementation. *Loop-tail* mice provide a useful model for research on the molecular interactions involved in the development of open and closed NTDs and for the design of prevention strategies for these diseases.

## Teaser

**Summary:** *Loop-tail* mice develop closed caudal neural tube defects and present cellular aggregates that may facilitate the sealing of these defects, and can thus be used as a model to study these congenital malformations.

## INTRODUCTION

Congenital malformations are the most common cause of infant death in developing countries and a major factor in the health problems of surviving babies. Neural tube (NT) closure or neurulation is a process that, in humans, occurs in the first month of pregnancy, the failure of which results in NT defects (NTDs). NTDs are the second most common cause of congenital malformations and affect 0.5 to two in every 1000 pregnancies worldwide (Mitchell, 2005). The different types of NTDs include lethal defects, in which the neural plate of most of the central nervous system or forebrain fails to form a tube (craniorachischisis and anencephaly, respectively), and caudal defects compatible with life, which feature near-complete closure of the NT (Copp et al., 2013; Wallingford et al., 2013).

In addition, there is a broad spectrum of skin-covered NTDs, including spina bifida occulta and spinal dysraphism, which constitute the least-defined group of NTDs and, as not all cases show symptoms, the actual incidence of which is also unclear. The most common form of close NTD is lumbosacral lipomyelomeningocele, resulting from the presence of lipomatous (fatty) tissue adherent to the dorsal medulla of the spinal cord, which emerges from a spinal defect together with the meninges of the spinal cord to form a posterior mass underneath the skin, usually in the lumbosacral region. Lipomyelomeningocele can manifest a wide variety of symptomatology including: cutaneous alterations (present in 90% of cases; Zerah et al., 2008); neurological deficits (present in 58% of patients; Rauzzino et al., 2001); sphincteric symptomatology (Zerah et al., 2008); and orthopaedic symptomatology, mainly represented by atrophy of the lower limb musculature (Zerah et al., 2008). From an embryological point of view, the developmental origin of closed NTDs is still poorly understood due to the lack of animal models, although numerous theories have been proposed throughout the history of medicine (reviewed by Copp et al., 2013; Zerah et al., 2008).

Owing to the complexity and difficulty of studying NTDs in humans, molecular and cellular mechanisms are often studied in animal models. It was precisely because of a variety of animal models that it was possible to demonstrate that the Wnt-planar cell polarity (PCP) pathway was among the signalling pathways involved in the neurulation process, and that its function was conserved across different vertebrate species (Wallingford, 2012). The Wnt-PCP pathway modulates the actin cytoskeleton and convergent extension cell movements, both of which are essential for neural plate folding and NT closure (López-Escobar et al., 2018; Ybot-González et al., 2007). *Loop-tail* (*Lp*) mice carry a mutation in the *Vangl2* gene, which encodes a transmembrane protein belonging to the core proteins of the Wnt-PCP pathway (Kibar et al., 2001; Murdoch et al., 2001). Homozygous mutant embryos, *Vangl2^Lp/Lp^*, show 100% penetrance of the NTD craniorachischisis. In the caudal neural plate, we have previously described how Wnt-PCP signalling organises apical F-actin together with myosin to drive neural plate folding through apical constriction, as well as providing neural folds with a rigid apical structure that endures the external forces required for their elevation (López-Escobar et al., 2018). In heterozygous mutants, *Vangl2^+/Lp^*, the disruption of the Wnt-PCP pathway increases the apical area of the cells within the neural plate, causing a loss of apical stiffness that affects neural fold morphology, hindering the elevation necessary for dorsal fusion. In our experience, all *Vangl2^+/Lp^* embryos show a wider posterior neuropore and an outward folding of the neural folds, affecting the correct formation of the caudal neural ectoderm before NT closure. Although these events induce an overall delay in the completion of NT closure at the caudal end of the embryo, only 6% of these embryos develop open spina bifida (López-Escobar et al., 2018).

Currently, there is no treatment that can effectively reverse the NTD phenotype once it has developed, so research in this field has focused on prevention strategies. The adequate supply of nutrients provided by the maternal diet is crucial for correct development of the embryo, and it is through this mouldable environmental factor that the phenotype of the embryo can be influenced. NTDs represent a type of congenital malformation that can be prevented by maternal dietary supplementation. Through prenatal prevention strategies with folic acid (FA) supplementation, the incidence of NTDs has drastically decreased the recurrent risk in the general population, reaching 70% for women supplemented with high levels of FA following a previous NTD pregnancy (De Wals et al., 2007; MRC Vitamin Study Research Group, 1991). However, these figures also reveal that this nutrient is not always effective in the prevention of NTDs, the so-called FA resistance (Bupp et al., 2015; Mosley et al., 2009; MRC Vitamin Study Research Group, 1991). For instance, maternal FA supplementation has not changed the incidence of lipomyelomeningocele (De Wals et al., 2008; Forrester and Merz, 2004; McNeely and Howes, 2004).

Here, to better understand the effect of *Vangl2* mutation on the morphological development of the caudal central nervous system, we analysed mouse embryos from developmental stages in which the NT is in a post-closure stage, at embryonic days (E) 10.5-14.5. We observed that most *Vangl2^+/Lp^* embryos developed caudal NTDs that were macroscopically undetectable as they were covered by non-neural ectoderm epithelium, and therefore fell into the category of closed NTDs. Our histological studies showed that the failure of dorsal fusion was ‘sealed’ by the formation of a cellular aggregate that resembled a lipomyelomeningocele. We present a mouse model of closed NTD that could possibly be considered a primordial lipoma, in which we have been able to study in detail its aetiology, so far little studied. Furthermore, an additive genetic interaction between *Vangl2* and *Daam1* was observed, as similar but more severe defects occurred in embryos in which both Wnt-PCP pathway genes were mutated. The *Vangl2^Lp^* allele was also found to interact with the hypomorphic allele of *Grhl3* to cause a more severe closed NTD but shifted to more anterior areas of the NT. We have also demonstrated that, as in human lipomyelomeningocele, closed NTDs in this *Vangl2* mouse model are resistant to maternal FA supplementation. Part of our work also involved developing analytical tools, as well as a systematic protocol to assess the severity of damage (supplementary Materials and Methods), which we hope will be of great use to researchers working on NTD prevention strategies.

## RESULTS

### *Vangl2^+/Lp^* embryos display a high incidence of dorsal fusion failure

**Fig. 1.**
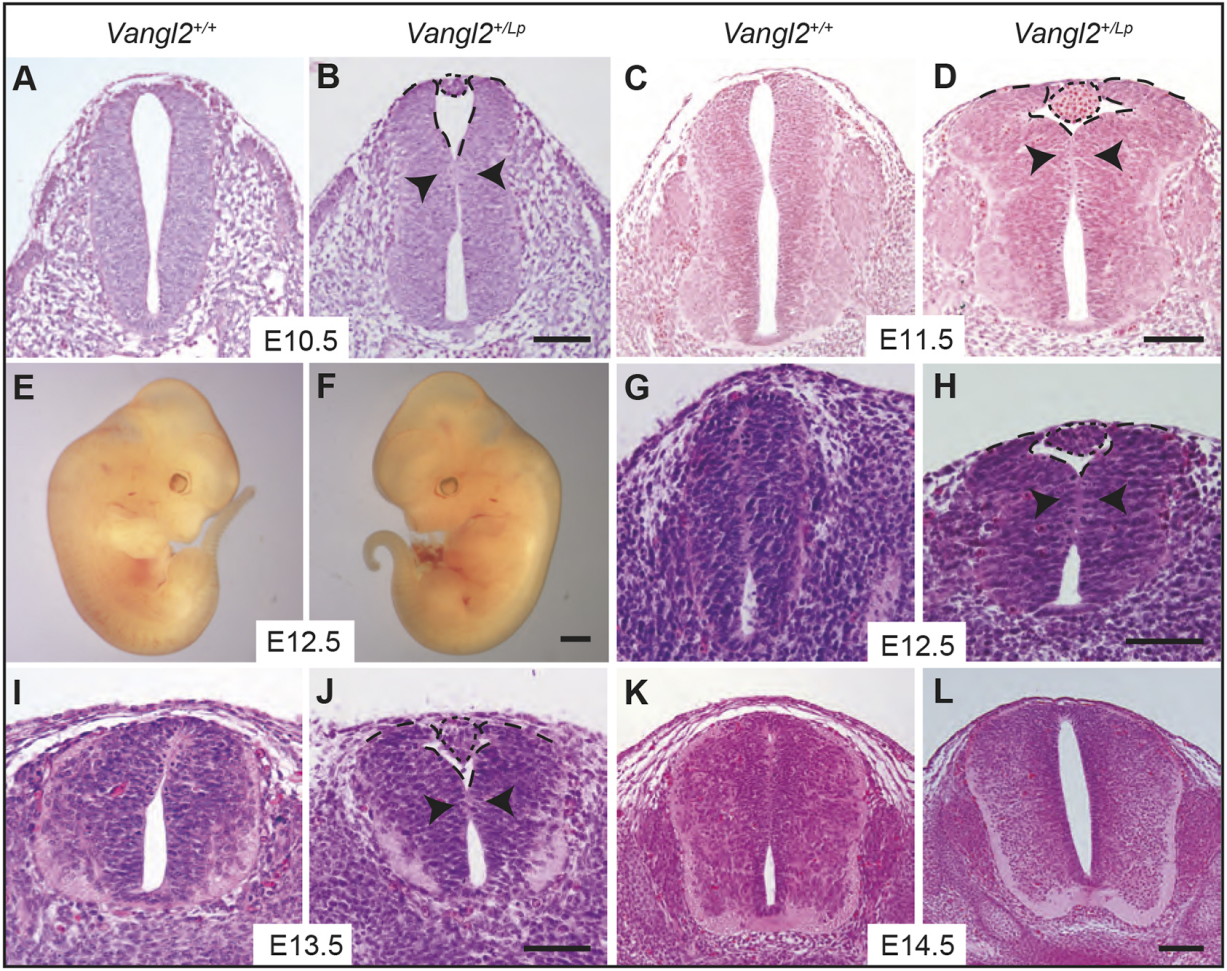
**Failure of dorsal neural tube fusion promotes CA formation in *Vangl2^+/Lp^* embryos.** (A-D,G-L) Transverse sections stained with Haematoxylin and Eosin through the lumbar region of *Vangl2^+/+^* (A,C,G,I,K) and *Vangl2^+/Lp^* (B,D,H,J,L) embryos at the indicated developmental stages (E10.5-E14.5). The short-dashed lines delimit the CAs. The long-dashed lines delimit the dorsal tip of the opposing neural folds, highlighting the failure of NT dorsal fusion of *Vangl2^+/Lp^* embryos at these post-fusion stages. Arrowheads indicate outward flexion of the neural folds. (E,F) Whole *Vangl2^+/+^* (E) and *Vangl2^+/Lp^* (F) mouse embryos at E12.5. Images are representative of *n*=8 E10.5, *n*=24 E11.5, *n*=18 E12.5, *n*=15 E13.5 and *n*=7 E14.5 embryos. Scale bars: 100 μm (A-D,G-J); 200 μm (K,L); 1000 μm (E,F).

Previous studies by our group showed that the caudal neuroectoderm cells of E9.5 *Vangl2^+/Lp^* embryos had an altered distribution of the apical cytoskeleton. This malformation had a direct impact on the folding processes of the caudal neural plate, as well as on the general morphology of the posterior neuropore (López-Escobar et al., 2018). To find out whether these alterations at early stages of neurulation have implications later in development, we decided to histologically analyse embryos at E10.5-E12.5, a developmental stage at which the *Vangl2^+/+^* NT is completely closed. This study revealed that whereas *Vangl2^+/+^* embryos showed a completely normal NT fusion (Fig. 1A,C,G), in *Vangl2^+/Lp^* embryos, the malformations of the neural folds became more pronounced with age, inducing an exacerbated outward flexion of the neural folds (Fig. 1B,D,H). A direct consequence of this aberrant bending was the separation of the dorsal tips of the neural folds, thus preventing their dorsal fusion, as evidenced in E10.5 embryos, in which delayed NT closure was observed for *Vangl2^+/Lp^* (López-Escobar et al., 2018). This fusion failure was histologically confirmed in a substantial number of embryos (E10.5, 4/8; E11.5, 17/24; and E12.5, 13/18), whereas it could not be observed macroscopically (Fig. 1E,F) as it was sealed by the appearance of a cellular aggregate (CA) that may have facilitated the covering of the defect by the surface ectoderm (Fig. 1B,D,H). This may explain why this defect has not been previously reported. To further investigate this caudal defect and the presence of the CA, we analysed older embryos. To our surprise, although the defect and CA were still visible at E13.5 (11/15; Fig. 1J), no CAs were visible in the caudal region at E14.5 (0/7; Fig. 1L) and the failure of dorsal fusion appeared to have been repaired or healed. At all ages tested, there was no evidence of CAs in *Vangl2^+/+^* embryos (*n*=33; Fig. 1A,C,G,I,K).

**Fig. 2.**
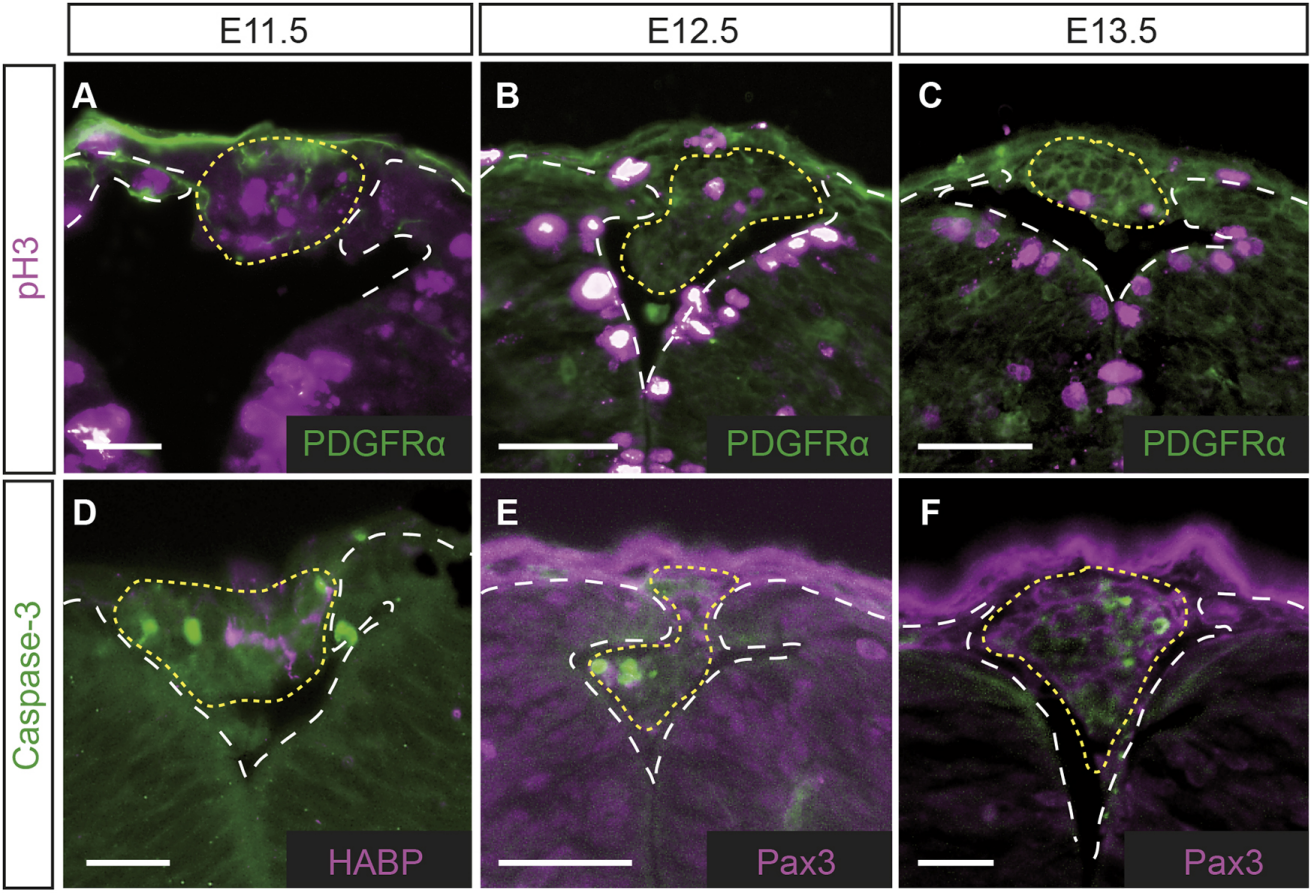
**Detection of cell proliferation and apoptosis in the cells within the CA.** (A-F) Transverse sections of CAs from *Vangl2^+/Lp^* embryos immunolabelled for phospho-histone 3 (pH3) and platelet-derived growth factor receptor α (PDGFRα) (A-C), caspase-3 and hyaluronic acid-binding protein (HABP) (D), or caspase 3 and Pax3 (E,F). The indicated double immunolabelling has been included to appreciate tissue contour. The yellow dotted lines delimit the CAs. The white dashed lines delimit the dorsal tip of the opposing neural folds, highlighting the failure of NT dorsal fusion of *Vangl2^+/Lp^* embryos at these post-fusion stages. Images are representative of at least *n*=3 embryos per study. Scale bars: 25 μm (A); 50 μm (B-F).

Given that the CA appears to fill the gap caused by dorsal fusion failure, to better understand the progression of this defect, we decided to study the cellular dynamics of the CA over time. Immunohistochemical analysis was performed at stages E11.5-E13.5 with phospho-histone 3 (pH3), a marker of cell proliferation (Fig. 2A-C). Cell division could be observed within the CA in *Vangl2^+/Lp^* embryos at E11.5 (Fig. 2A; *n*=3) and this expression was maintained at E12.5 (Fig. 2B; *n*=6) and E13.5 (Fig. 2C; *n*=3), suggesting that the CA continued to grow at least until E13.5. The absence of the CA at E14.5, together with the fact that in some CAs, a cluster of cells appeared to detach and fall into the lumen of the NT (Fig. 5; see ‘Characteristics and fate of the CAs’ section below), prompted us to examine apoptosis by analysing caspase-3 expression by immunofluorescence at stages E11.5-E13.5 (Fig. 2D-F; *n*=3). The results showed the existence of apoptosis in the CA at all these three stages. However, the comparable levels of cell proliferation and apoptosis observed at the three stages analysed suggested that neither does cell proliferation appear to play a significant role in the appearance of CAs, nor can apoptosis alone explain the disappearance of CAs at later stages.

### Molecular profile of the cells that constitute the CA

**Fig. 3.**
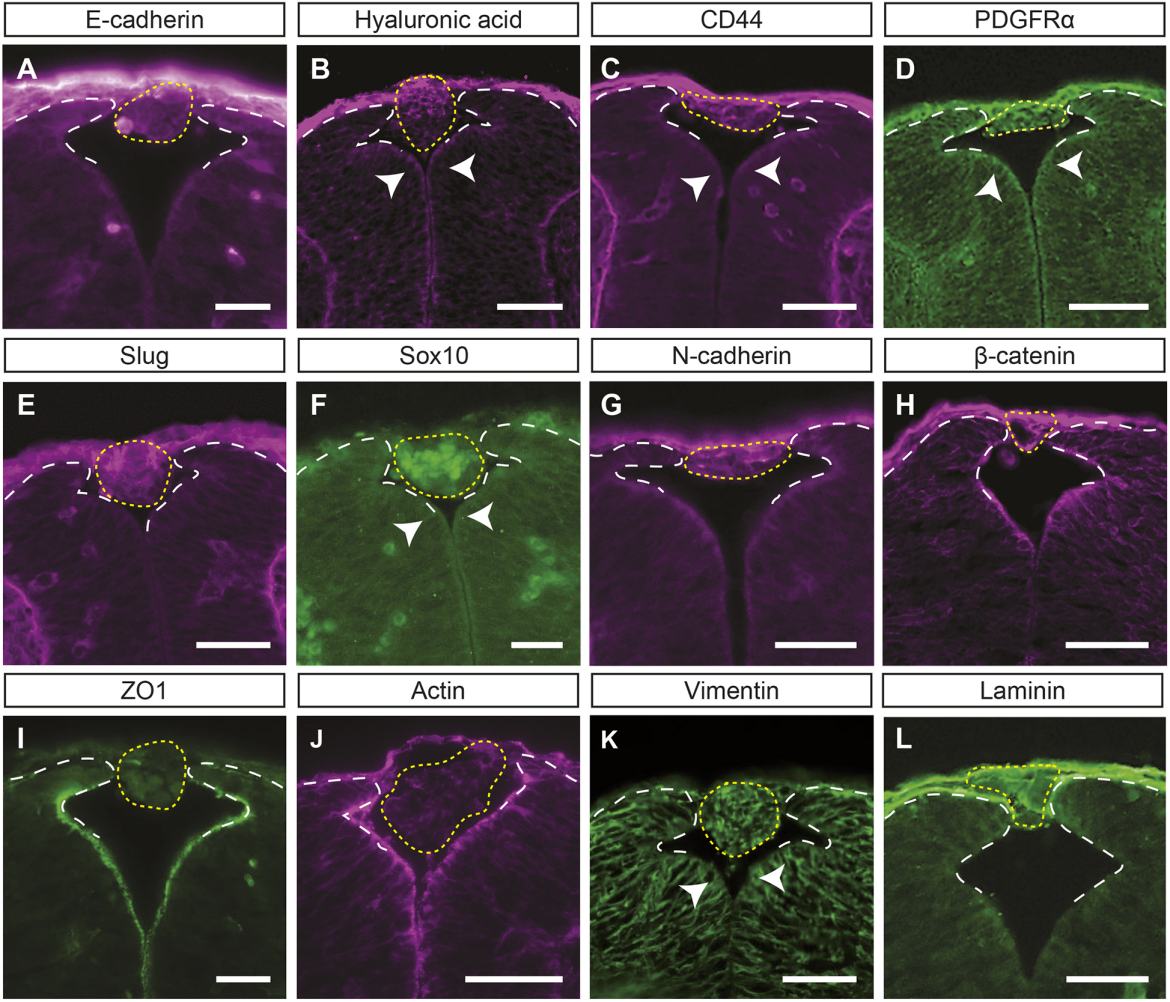
**Insights into the molecular profile of the CAs.** (A-L) Transverse sections of CAs from E12.5 *Vangl2^+/Lp^* embryos immunolabelled for E-cadherin (A), CD44 (C), PDGFRα (D), Slug (E), Sox10 (F), N-cadherin (G), β-catenin (H), ZO1 (I), vimentin (K) and laminin (L). Hyaluronic acid (B) and actin (J) distribution were visualised in homologous transverse sections of CAs using HABP and phalloidin, respectively. The yellow dotted lines delimit the CAs and the white dashed lines mark the dorsal tip of the opposing neural folds, highlighting the failure of NT dorsal fusion of *Vangl2^+/Lp^* embryos at these post-fusion stages. Arrowheads indicate outward flexion of the neural folds. Images are representative of at least *n*=3 embryos per study. Scale bars: 50 μm.

Before embarking on a more in-depth analysis of the CA, we decided to explore its molecular characteristics, as this would allow us to design more specific tools for further studies. As a main approach to describe the molecular profile of the CA, we analysed it by immunolabelling, using molecular markers from different tissues surrounding the CA at E11.5-E13.5, the developmental window during which CAs were observed (Fig. 3; Figs S1-S3).

Firstly, we thought that the cells may originate from an event similar to wound healing, which, as we have described previously, uses the same molecular machinery as the caudal NT to achieve closure (Fernández-Santos et al., 2021). Thus, we hypothesised that the closure process, owing to morphological abnormalities in the *Vangl2^+/Lp^* neural folds, may be more fragile and needs to be enhanced by the wound healing process. To this end, immunolabelling against the epithelial marker E-cadherin (CDH1) revealed that the only E-cadherin-positive cells observed in *Vangl2^+/Lp^* E12.5 embryos were located in the surface ectoderm (Fig. 3A; *n*=5), but no staining was detected inside the CA; a similar pattern was also observed at E11.5 and E13.5, which did not seem to differ from the E-cadherin expression of wild-type *Vangl2^+/+^* E11.5 embryos (Fig. S1A-C). We know from our previous work that during embryonic wound healing in the caudal area of the NT, there is an increased expression of hyaluronic acid (HA), the HA receptor CD44 and platelet-derived growth factor receptor α (PDGFRα, encoded by *Pdgfra*; Fernández-Santos et al., 2021). Here, as in an induced wound, we observed HA, CD44 and PDGFRα expression in the CA of *Vangl2^+/Lp^* embryos at E11.5-E13.5 (*n*=3), suggesting a localised activation of the healing mechanism in the caudal-most closure zone and/or reinforcement from the surface epithelium to complete closure. The detection of this localised activation appears to be specific to *Vangl2^+/Lp^* embryos as there was no evidence of HA [detected by HA-binding protein (HABP)], CD44 or PDGFRα in the dorsal area in *Vangl2^+/+^* embryos that does not correspond with the surface ectoderm (Fig. 3B-D; Fig. S1D-L).

Taking into account that mouse neural crest cells (NCCs) migrate just after the caudal NT closes, we hypothesised that part of these cells, after undergoing the epithelial-mesenchymal transition (EMT) and acquiring a migratory status (Lamouille et al., 2014), could migrate into the gap between the dorsal folds. In this context, we observed that NCC-specific proteins such Slug (encoded by *Snai2*) (Fig. 3E; *n*=3) and Sox10 (Fig. 3F; *n*=3), the expression of which is required to activate the EMT programme during development (Steventon et al., 2005), were expressed by cells within the CA. The presence of the mesenchymal marker N-cadherin (CDH2) was also detected (Fig. 3G; *n*=3), consistent with the so-called cadherin switch, the downregulation of E-cadherin followed by upregulation of N-cadherin, a process that facilitates cell migration (Wheelock et al., 2008). We also observed the presence of β-catenin (CTNNB1) at cell-cell junctions in CAs (Fig. 3H, *n*=3), suggesting that it may be acting through its adhesive role, potentially working together with N-cadherin (Valenta et al., 2012). Following NCC differentiation events, we observed the absence of ZO1 (TJP1) and apical actin in the CAs (Fig. 3I,J; *n*=3, respectively), suggesting a loss of apical-basal polarity, as cells undergoing EMT reorganise their cortical actin cytoskeleton into one that allows directional motility and change their intermediate filament composition from cytokeratin to vimentin. The acquisition of vimentin intermediate filaments (Fig. 3K; *n*=3) in the CAs was also detected. The presence of laminin was also studied, as it is a major component of the basement membrane with an important role in NCC migration (Duband and Thiery, 1987; Kalcheim, 2018; Perris and Perissinotto, 2000). Laminin appeared to be present without a clear pattern within the CAs, which supported the idea of polarity loss (Fig. 3L; *n*=3). Knowing the transience of the molecular composition of the NCCs, we corroborated that the expression of the aforementioned markers was comparable in the developmental window during which CAs are observed, E11.5-E13.5. Moreover, this pattern of expression appeared to be specific to *Vangl2^+/Lp^* embryos as no expression was detected in *Vangl2^+/+^* embryos (Fig. 3E-L; Figs S2 and S3).

### Characteristics and fate of the CAs

**Fig. 4.**
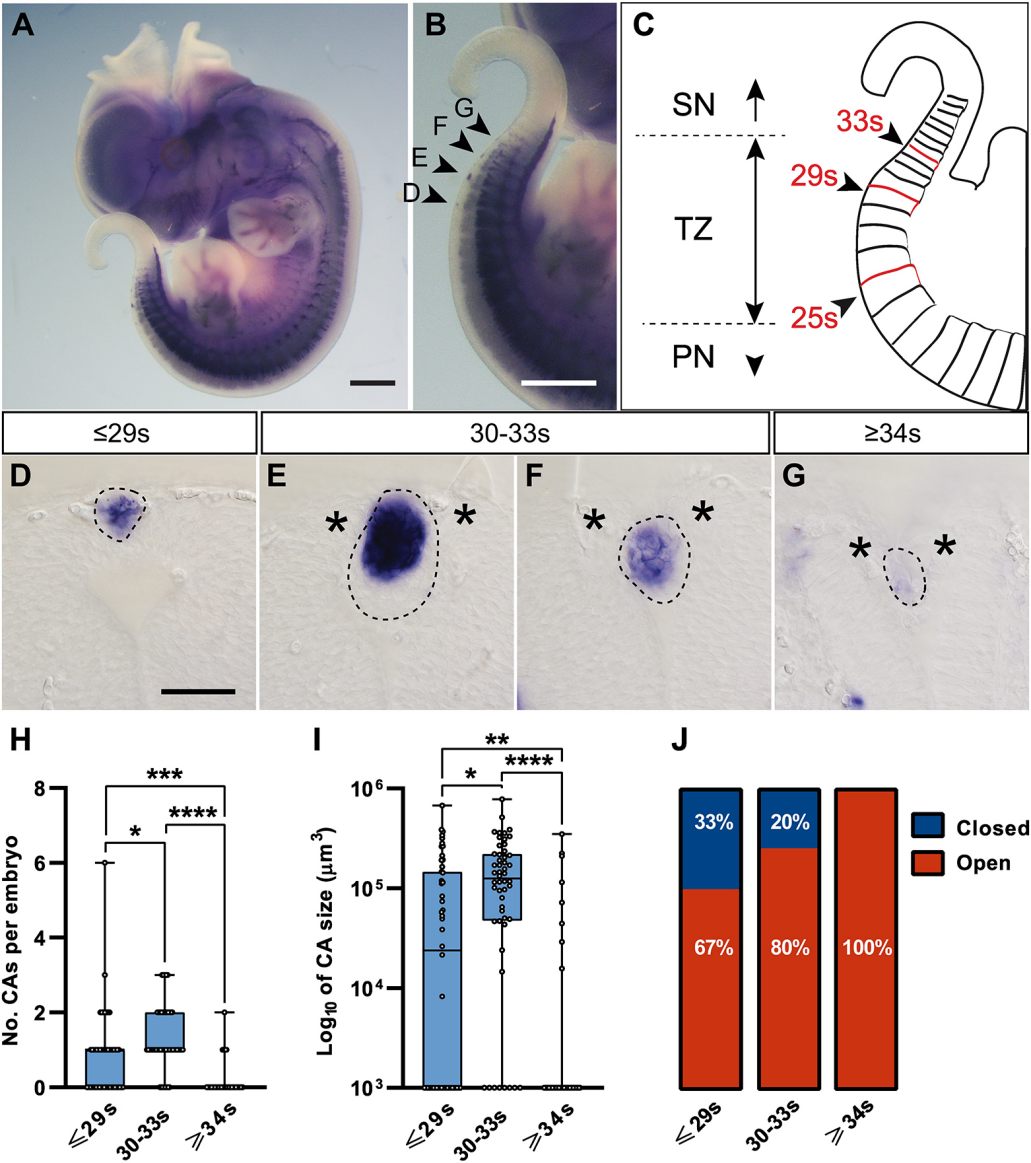
***Sox10* mRNA expression and characteristics of the CAs in E12.5 *Vangl2^+/Lp^* embryos.** (A) Representative image of a whole embryo labelled by *in situ* hybridisation for *Sox10*. (B) Magnification of the caudal region of the embryo, where the CAs are located. (C) Diagram of the caudal region of the embryo marking relevant somites that define the transition zone (TZ) between primary neurulation (PN) and secondary neurulation (SN) in *Vangl2^+/Lp^* embryos. (D-G) Transverse sections through the CAs at specific levels of the anteroposterior axis of the embryo indicated with the corresponding somite number and marked with arrowheads in B: in the ≤29s zone (D), in the 30-33s zone (E,F) and in the ≥34s zone (G). Dashed lines delimit the CAs and asterisks at the dorsal tips of neuronal folds highlight the failure of NT dorsal fusion of *Vangl2^+/Lp^* embryos at these post-fusion stages. Scale bars: 1000 µm (A,B); 50 μm (D-G). (H,I) Number (H) and size (I) of the CAs found in each of the defined anteroposterior zones. Boxes show the 25-75th percentiles, whiskers show the minimum and maximum, and the median is marked with a line. Ordinary one-way ANOVA followed by Tukey's multiple comparisons test was used for statistical analysis; *P<0.05, **P<0.01, ***P<0.001, ****P<0.0001. (J) Percentage of the CAs associated with an open or closed NT in each zone. A total of *n*=52 E11.5-E13.5 *Vangl2^+/Lp^* embryos were analysed.

**Fig. 5.**
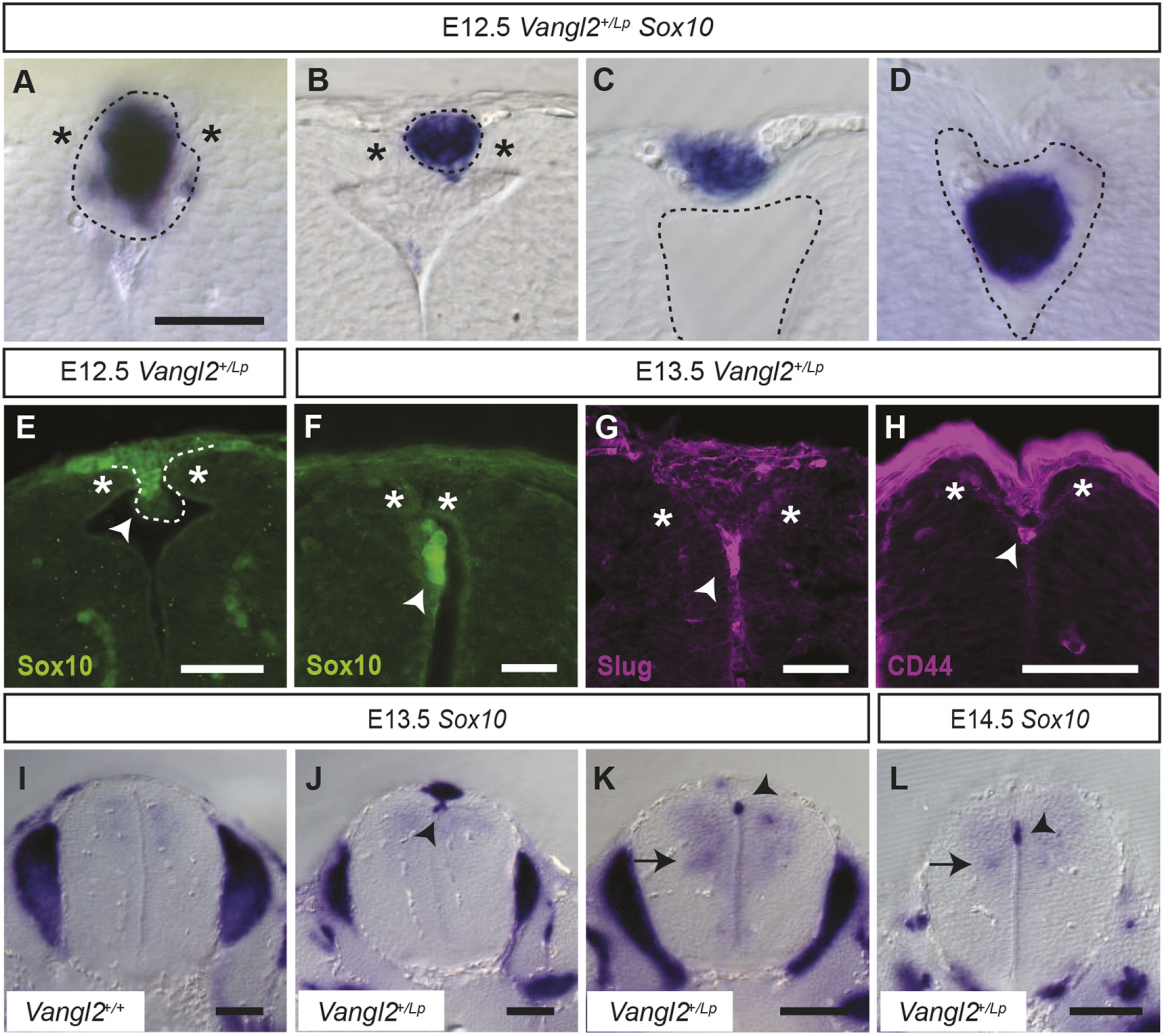
**Variability in the morphology of the CAs and their possible fate.** (A-D) Transverse sections of CAs from different E12.5 *Vangl2^+/Lp^* embryos labelled by *in situ* hybridisation for *Sox10*: a large CA (A), a small CA (B), a CA above fused NT (C) and a CA below fused NT (D). (E-G) Transverse sections of E12.5-E13.5 embryos immunolabelled for Sox10 (E,F), Slug (G) and CD44 (H). (I,J) *In situ* hybridisation for *Sox10* at E13.5 in *Vangl2^+/+^* (I) and *Vangl2^+/Lp^* embryos (J,K) and at E14.5 in a *Vangl2^+/Lp^* embryo (L). The dotted lines in A,B,E delimit the contour of the CAs in *Vangl2^+/Lp^* embryos, and in C,D delimit the dorsal closed NT. Arrowheads in E-H,J-L indicate cells of the CA falling into the NT lumen. Asterisks in A,B,E-H at the dorsal tips of neuronal folds highlight the failure of NT dorsal fusion of *Vangl2^+/Lp^* embryos at these post-fusion stages. Black arrows in K,L indicate the overexpression of *Sox10* within the NT. Scale bars: 50 μm (A-H); 100 µm (I-L).

Once the expression of NCC genes by CA cells was determined, we next designed the tools to further characterise the phenotype observed in *Vangl2^+/Lp^* embryos. For accurate characterisation of the CAs, we decided to analyse whole embryos by *in situ* hybridisation for *Sox10*, as it is a marker for NCCs and our immunostaining results showed a strong presence of this transcription factor in the CAs (Fig. 3F). The expected pattern of *Sox10* expression in the dorsal root and in sympathetic and cranial ganglia (Matera et al., 2008) was observed in all embryos regardless of their genotype (Fig. 4A). In *Vangl2^+/Lp^* embryos, *Sox10* also marked CAs on the dorsal side of the caudal NT, which could be clearly identified as the NCCs at this anteroposterior level had already migrated away from the dorsal region of the NT, as defined in the *Vangl2^+/+^* littermates (Fig. 5I; Fig. S2D). The study of whole embryos revealed that most embryos developed more than one CA (Fig. 4B) and these were located within three specific zones (Fig. 4C): the zone anterior to somite 29 (≤29s; Fig. 4D); the intermediate zone, between somites 30 and 33 (30-33s; Fig. 4E,F); and the caudal zone, posterior to somite 34 (≥34s; Fig. 4G). As we have previously observed (López-Escobar et al., 2018), the region where CAs are found in *Vangl2^+/Lp^* embryos corresponds to the transition zone between primary and secondary neurulation (Fig. 4C).

Another aspect that drew attention was the variability of sizes, as within the same embryo, we could find CAs of different dimensions (Fig. 4D-G, Fig. 5A,B). Therefore, taking into account that the CA appears to be formed as a ‘filler’ of the gap left by the dorsal fusion failure, we hypothesised that the number of CAs likely corresponds to the number of NT regions affected per embryo, and the size of the CA may be directly related to the size of the NT affected by the fusion failure. Hence, a larger size of the CAs may imply a greater severity of the defect. We noticed that the intermediate anteroposterior zone (30-33s) of the NT seemed more susceptible to closure failure, as it presented a higher CA number and size than the ≤29s and ≥34s zones (Fig. 4H,I).

In addition, we also observed CAs associated with a closed NT, either below or above the NT (Fig. 5C,D), suggesting a late closure. To corroborate this idea, we analysed the percentage of CAs associated with closed NTs as development progressed. CAs were analysed histologically in ten embryos at E11.5, 32 embryos at E12.5 and ten embryos at E13.5. This process appeared to follow both an anteroposterior progression (Fig. 4J) as well as a temporal progression, as 0% of CAs (0/15) were associated with a closed NT at E11.5, and this percentage gradually increased at later stages: 18% (11/61) at E12.5 and reaching 60% (15/25) at E13.5. These data indicate that it is likely that the presence of the CA and the sealing of the defect by the superficial ectoderm may facilitate the late progression of NT closure.

After these observations, we wondered what the fate of the CA cells was, given that at E14.5, we could not observe any CAs (Fig. 1L). Upon examination of sections of stages E12.5-E13.5, we observed that occasionally cells from the ventral part of the CA detached and fell into the lumen. Some of these continued to express NCC markers (such as Slug and Sox10) or CD44 (Fig. 5E-H). These observations could be verified by *in situ* hybridisation sections for *Sox10* at stages E13.5-E14.5, where it was not only seen that there were CA cells falling into the lumen (Fig. 5J-L), but also that these cells somehow could be responsible for an increase in the normal *Sox10* expression in the NT (Fig. 5K,L).

Taken together, these results suggest that in *Vangl2^+/Lp^* embryos, once the NCCs start migrating towards their future destination, some of the cells are trapped in the gap produced by the failure of NT dorsal fusion, forming the CA. The presence of this CA may facilitate the surface ectoderm to cover the closure failure. In addition, our results suggest that in *Vangl2^+/Lp^* embryos, owing to the structural fragility caused by the altered distribution of actin microfilaments, the fusion of the transition zone between primary and secondary neurulation appears to be particularly fragile.

### Assessment of the severity of dorsal fusion failure associated with the CA in *Vangl2^+/Lp^* embryos

We next designed a protocol to assess several parameters associated with the caudal damage observed in *Vangl2^+/Lp^* embryos, based on *Sox10 in situ* hybridisation. With this protocol, we assessed the severity of the caudal NT fusion failure based on the following parameters: (1) incidence, the percentage of embryos in a given condition that present CAs; (2) number, (3) anteroposterior location and (4) size of the CAs, as readouts of the severity of the dorsal NT fusion failure; (5) percentage of CAs associated to a closed NT, as an indicator of the late progression of NT closure; and (6) tail curvature, a parameter that has been previously associated with caudal damage (De Castro et al., 2018; see supplementary Materials and Methods for further details). During the course of this analysis, we found that both the number and size of CAs did not change significantly between E11.5 and E13.5, so it seemed appropriate to pool the results of these three stages (Fig. S4). We also found that the severity of the phenotype was independent of the litter, as both embryos with mild and severe defects were found in the same litter (*n*=14 litters analysed).

#### Incidence

The presence of CAs was observed at stages E11.5-E13.5, as shown by histology (Fig. 1). With *Sox10 in situ* hybridisation, a 100% incidence of this phenotype was observed, as all embryos had one or more CAs (Fig. 4; Table S1; *n*=52). The slight discrepancy with the histological results could simply be due to the higher sensitivity to detect CAs when performing *Sox10 in situ* hybridisation at the whole embryo level. The *in situ* hybridisation results also corroborated the absence of CAs at E14.5 (0/6 embryos).

#### Number of regions affected by NT fusion failure

We measured the number of CAs per embryo as a readout of the number of regions affected by NT fusion failure. *Vangl2^+/Lp^* embryos presented an average (±s.d.) of 2.2±1.2 CAs, with a maximum of six CAs per embryo (Table S1; *n*=52).

#### Location of NT fusion failure

The CAs found in *Vangl2^+/Lp^* embryos presented the following distribution according to the three anteroposterior zones defined earlier: 36% of the CAs were in the ≤29s zone (42/116 CAs) with an average of 0.8±1.1 CAs/embryo; 57% of the CAs were in the 30-33s zone (66/116) with 1.3±0.8 CAs/embryo; and 7% of the CAs were in the ≥34s zone (8/116) with 0.2±0.4 CAs/embryo (Fig. 4H; Table S2). For this parameter, 0 was considered when an embryo did not present any CAs in a given zone. The data suggest that the presence of CAs in the most anterior level, ≤29s, may be associated with a more severe dorsal fusion failure, as it was observed mainly in embryos with a high number of CAs (one CA: 2/17, 12%; two CAs: 10/17, 59%; three CAs: 7/9, 78%; four CAs: 8/8, 100%; six CAs: 1/1, 100%).

#### NT affected by dorsal fusion failure

Given that the CA seemed to fill the space left by a dorsal fusion failure, we measured CA size as a readout of NT damage. The average (±s.d.) CA size in *Vangl2^+/Lp^* embryos was 2.74±1.83×10^5^ µm^3^ (Table S1; *n*=52). It was noteworthy that the CA was significantly bigger in the 30-33s region, suggesting a more severe dorsal fusion failure in the intermediate anteroposterior zone: ≤29s zone, 0.94±1.39×10^5^ µm^3^; 30-33s zone, 1.60±1.52×10^5^ µm^3^; ≥34s zone, 0.20±0.65×10^5^ µm^3^ (Fig. 4I; Table S2). For this parameter, 0 was considered when an embryo did not present any CAs in a given zone.

#### Late NT closure progression

We found that some CAs were below/over a closed NT, indicating that although CAs formed where the NT had failed to fuse, the presence of the CA may have facilitated late closure, thereby attenuating dorsal damage. In E11.5-E13.5 *Vangl2^+/Lp^* embryos, we found that 33% of the CAs located in anterior positions (≤29s) were associated with a closed NT (14/42), whereas in more caudal positions, this figure was clearly reduced (20% in zone 30-33s, 13/66; 0% in zone ≥34s, 0/8; Fig. 4J; Table S2).

#### Tail curvature

One of the most obvious characteristics of *Vangl2^+/Lp^* mutants is the presence of a curled tail. As the tail grows and curls over time, including the timeframe of our study, we only measured this parameter at E12.5. *Vangl2^+/Lp^* embryos had a mean curvature of 1.56±0.41 mm^−1^ (*n*=31; Table S1).

### Dorsal fusion failure associated with the CA of *Vangl2^+/Lp^* embryos in combination with mutant alleles of other NTD genes

Having established the aetiology of the closed NTD caused by a specific genetic pathway (Wnt-PCP) and a protocol for assessing the severity of dorsal damage specific to this NTD (supplementary Materials and Methods), we next decided to evaluate this protocol in possible detrimental scenarios. To do so, we used double mutants of *loop-tail* and we analysed (1) a more severe blockade of the Wnt-PCP pathway (*Daam1* mutant) and (2) the effects of deregulation of the Wnt-PCP pathway in combination with a mutation in another gene (*Grhl3*) that is associated with caudal NTDs but is independent of the Wnt-PCP pathway.

#### Phenotypic evaluation of caudal NT fusion failure in double mutants of members of the Wnt-PCP pathway

**Fig. 6.**
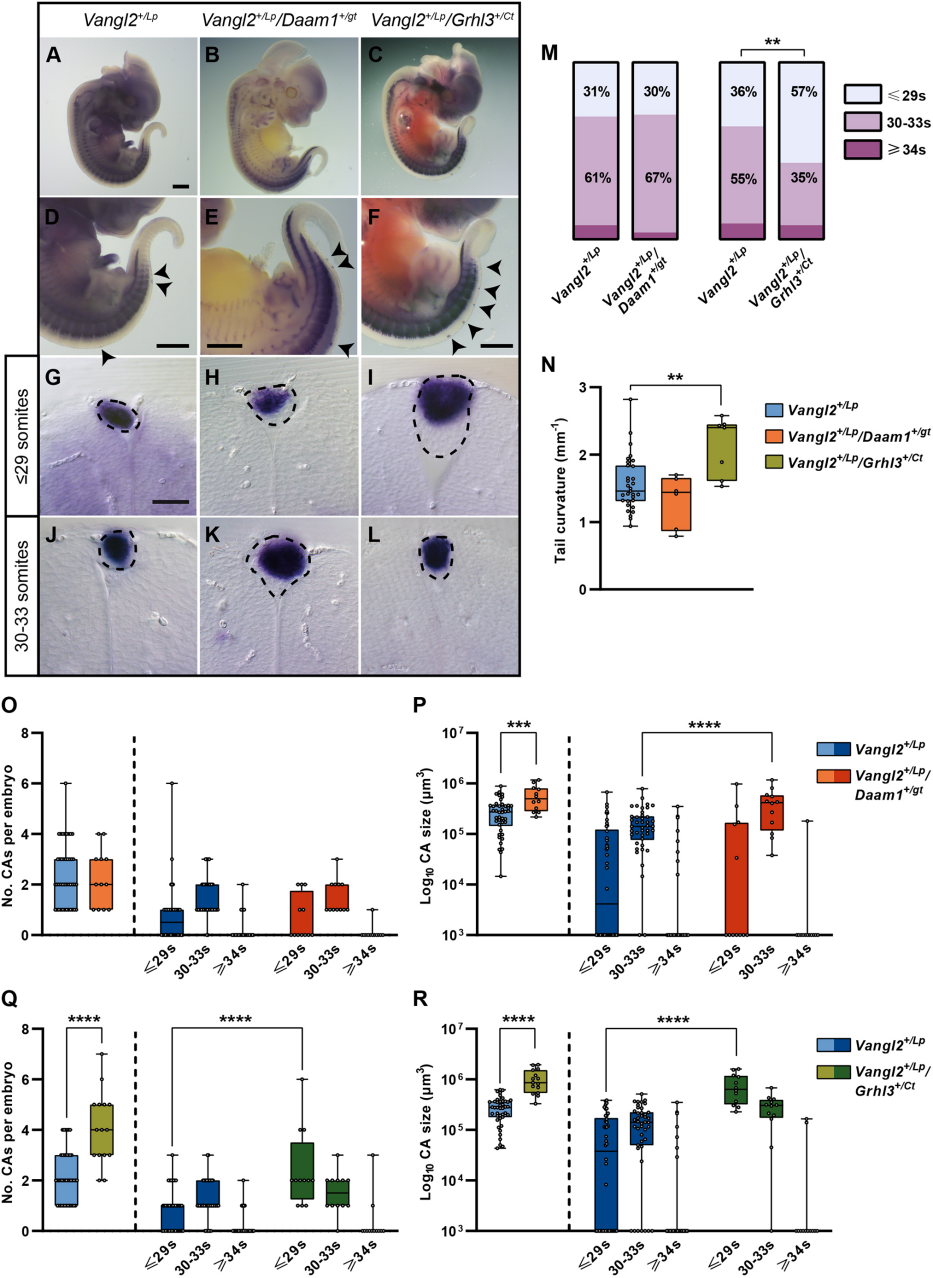
**Detection of more severe dorsal fusion failure in double mutants of NTD-associated genes.** (A-C) *In situ* hybridization for *Sox10* in E12.5 whole embryos: *Vangl2^+/Lp^* (A), *Vangl2^+/Lp^/Daam1^+/gt^* (B) and *Vangl2^+/Lp^/Grhl3^+/Ct^* (C). (D-F) Magnification of the caudal region of the embryos in A-C, indicating the location of CAs by black arrowheads. (G-L) Transverse sections through the CAs located at the most anterior level of somites ≤29 (G-I), and at the level of somites 30-33 (J-L). The dashed black lines define the CAs. Images are representative of *n*=42 *Vangl2*^*+/Lp*^, *n*=12 *Vangl2*^*+/Lp*^*/Daam1*^*+/gt*^ and *n*=15 *Vangl2*^*+/Lp*^*/Grhl3*^*+/C*^ embryos. Scale bars: 1000 μm (A-F); 50 μm (G-L). (M) Distribution of the CAs according to the defined anteroposterior zones in each of the genotypes studied. (N) Curvature of the tail in E12.5 embryos for the three genotypes studied. (O,P) Number (O) and size (P) of the CAs found in E12.5-E13.5 *Vangl2^+/Lp^* and *Vangl2^+/Lp^/Daam1^+/gt^* embryos. (Q,R) Number (Q) and size (R) of the CAs found in E11.5-E12.5 *Vangl2^+/Lp^* and *Vangl2^+/Lp^/Grhl3^+/Ct^* embryos. In O-R, the boxplots on the left (light colours) represent the total embryo data, whereas the boxplots on the right (dark colours) show the data in each of the defined anteroposterior zones. Boxes show the 25-75th percentiles, whiskers show the minimum and maximum, and the median is marked with a line. Statistical analyses: χ^2^ test (M); ordinary one-way ANOVA with Dunnett's post hoc test (N); unpaired two-tailed *t*-test for the comparisons between genotypes and two-way ANOVA followed by Šídák's multiple comparisons test for the comparisons per zones (O-R). Only significant differences are shown: **P<0.01, ***P<0.001, ****P<0.0001.

Daam1 (Dishevelled-associated activator of morphogenesis 1) belongs to the formin family of actin nucleators, participates in the Wnt-PCP pathway (Habas et al., 2001; Sato et al., 2006), and has been associated with embryonic wound healing (Li et al., 2011). To further investigate whether the phenotype under study in *Vangl2^+/Lp^* embryos (Fig. 6A,D,G,J) could be worsened by additional Wnt-PCP impairment, we assessed dorsal damage in *Vangl2^+/Lp^/Daam1^+/gt^* double-mutant embryos. These double mutants had previously shown an increased incidence of open spina bifida relative to that in *Vangl2^+/Lp^* (López-Escobar et al., 2018). Preliminary histological results from our laboratory showed that E12.5-E13.5 *Daam1^+/gt^* embryos did not develop CAs (0/13), nor did E14.5 *Vangl2^+/Lp^/Daam1^+/gt^* embryos (0/3).

In this initial histological evaluation, there appeared to be no differences in the phenotype of the double mutants with respect to *Vangl2^+/Lp^*. Therefore, we evaluated the phenotype in more detail by performing *in situ* hybridisation for *Sox10* and assessing the parameters described in our protocol (supplementary Materials and Methods; Fig. 6B,E,H,K). In order to relate the histological studies to the new *Sox10* expression analyses, age-matched double-mutant embryos were used: E12.5-E13.5 *Vangl2^+/Lp^/Daam1^+/gt^* embryos were analysed and compared to E12.5-E13.5 *Vangl2^+/Lp^* embryos. Our results showed that the only parameter affected in *Vangl2^+/Lp^/Daam1^+/gt^* embryos relative to *Vangl2^+/Lp^* embryos was the CA size, a readout of the NT area affected by dorsal fusion failure, which was significantly greater in the double mutants (5.78±3.17×10^5^ µm^3^, *n*=12, versus 2.87±1.94×10^5^ µm^3^, *n*=42; *P*=0.0002; Fig. 6P; Table S1). The most affected region in the double mutants was between somites 30 and 33, which showed a larger CA size compared with that in *Vangl2^+/Lp^* embryos (4.22±3.33×10^5^ µm^3^ versus 1.79±1.53×10^5^ µm^3^; *P*<0.0001). Both the ≤29s and ≥34s regions showed no significant differences relative to those in *Vangl2^+/Lp^* embryos (Fig. 6P; Table S2).

#### Phenotypic evaluation of caudal NT fusion failure in double mutants of two genes associated with spinal NTDs

Following a similar approach, we crossed *Vangl2^+/Lp^* with homozygous curly-tailed mice (*Grhl3^Ct/Ct^*), a hypomorphic *Grhl3* mutant associated to spinal NTDs caused by an altered balance of cell proliferation between the hindgut and neural plate (Gustavsson et al., 2007). Previous studies have shown that although *Vangl2^+/Lp^/Grhl3^Ct/Ct^* double mutants had a high incidence of open caudal defect, no such defect was found in *Vangl2^+/Lp^/Grhl3^+/Ct^* embryos (De Castro et al., 2018). Preliminary histological results in our laboratory showed that at E11.5-E12.5, the incidence of CAs was 20% (2/10) in *Grhl3^Ct/Ct^* embryos and 93% (13/14) in *Vangl2^+/Lp^/Grhl3^+/Ct^* embryos. As for the *Daam1* study, age-matched double-mutant embryos were used to relate histological and *Sox10 in situ* analyses; these were compared to E11.5-E12.5 *Vangl2^+/Lp^* embryos (Fig. 6C,F,I,L).

Our *Sox10 in situ* hybridisation results showed that none of the *Vangl2^+/+^/Grhl3^+/Ct^* embryos analysed (*n*=8) presented CAs, whereas *Vangl2^+/Lp^*/*Grhl3^+/Ct^* embryos (*n*=15) had a 100% incidence of CAs. *Vangl2^+/Lp^*/*Grhl3^+/Ct^* had significantly more CAs per embryo than their *Vangl2^+/Lp^* counterparts, suggesting that the number of regions affected by NT fusion failure may be higher in the double mutants (4.1±1.4 CAs/embryo, *n*=15, versus 2.2±1.0 CAs/embryo, *n*=42; *P*<00001; Fig. 6Q; Table S1). For instance, we found up to seven CAs per embryo in *Vangl2^+/Lp^*/*Grhl3^+/Ct^* embryos and only a small percentage (13%) of these double mutants had one or two CAs, whereas most (67%) *Vangl2^+/Lp^* embryos had one or two CAs (*P*<0.0001; Table S1).

The distribution of the CAs in the anteroposterior axis was also different in the double mutants: *Vangl2^+/Lp^*/*Grhl3^+/Ct^* embryos showed a significant increase in the number of CAs in the most anterior region (≤29s) compared to *Vangl2^+/Lp^* embryos (2.4±1.5 versus 0.8±0.8, respectively; *P*<0.0001; Fig. 6Q; Table S2). Moreover, 100% of the *Vangl2^+/Lp^*/*Grhl3^+/Ct^* embryos presented CAs in the anterior ≤29s zone (15/15) versus 57% of the *Vangl2^+/Lp^* embryos (24/42). This high incidence of CAs in anterior areas of the spine suggests a more anterior location of the NT fusion failure and may imply a more severe phenotype (Fig. 6M).

In addition to the differences in CA number and anteroposterior location, *Vangl2^+/Lp^/Grhl3^+/Ct^* embryos also presented a significant increase in CA size compared to that in *Vangl2^+/Lp^* embryos (10.13±5.20×10^5^ µm^3^, *n*=15, versus 2.79±1.51×10^5^ µm^3^, *n*=42; *P*<0.0001; Fig. 6R; Table S1). When analysing CA size per somite region, we found that this parameter was significantly larger in the most anterior ≤29s zone (7.75±4.87×10^5^ µm^3^ versus 0.96±1.20×10^5^ µm^3^; *P*<0.0001; Fig. 6R; Table S2), which suggests that the dorsal fusion failure of the NT is greater in *Vangl2^+/Lp^/Grhl3^+/Ct^* compared to that in *Vangl2^+/Lp^* embryos.

The last parameter that we analysed was the tail curvature. E12.5 *Vangl2^+/Lp^/Grhl3^+/Ct^* embryos presented higher mean curvature of the tail compared to that of E12.5 *Vangl2^+/Lp^* embryos (2.13±0.44 mm^−1^, *n*=7, versus 1.56±0.41 mm^−1^, *n*=31; *P*=0.0041; Fig. 6N; Table S1). We speculate that the greater severity of dorsal caudal damage observed in the anterior regions of *Vangl2^+/Lp^/Grhl3^+/Ct^* embryos, either in number of CAs or in affected NT region, could be associated with the greater curvature of the tail.

Overall, the results summarised in Fig. 6 and Tables S1 and S2 indicate that the closed NTD phenotype observed in *Vangl2^+/Lp^* embryos can deteriorate in combination with mutant alleles of other NTD genes. A double knockdown in the Wnt-PCP pathway, *Vangl2* and *Daam1*, impaired the most affected region in *Vangl2^+/Lp^* embryos (30-33s), without increasing the damage in the other two regions. However, the double mutant of *Vangl2* with *Grhl3*, an NTD-associated gene but independent of the Wnt-PCP pathway, increased damage in more anterior zones.

### Assessment of the potential preventive effect of maternal dietary supplementation on the development of *Vangl2^+/Lp^*-associated caudal NTDs

We then assessed the occurrence of caudal NT fusion failure in a potentially beneficial scenario, which would be for an embryo to develop under the influence of maternal dietary supplements known for their NTD-preventive properties: FA, *myo*-inositol (MI), and D*-chiro*-inositol (CI) (Greene and Copp, 2005). A single supplementation alone may not be sufficient as a preventive measure against a given NTD, but a combination of nutrients could provide benefits. Given that FA is a proven strategy for NTD prevention and that between MI and CI, the latter has been previously shown to be more effective (Cogram et al., 2002; Greene and Copp, 2005), we also tested a combination of FA and CI supplementation (FA+CI). These groups were compared with a control group fed a non-supplemented (NS) normal diet. To reduce possible alterations due to maternal genotype, the dams used for this study were *Vangl2^+/Lp^*, and embryos were collected at E12.5 to ensure that the period of exposure to each maternal supplementation was equivalent.

**Fig. 7.**
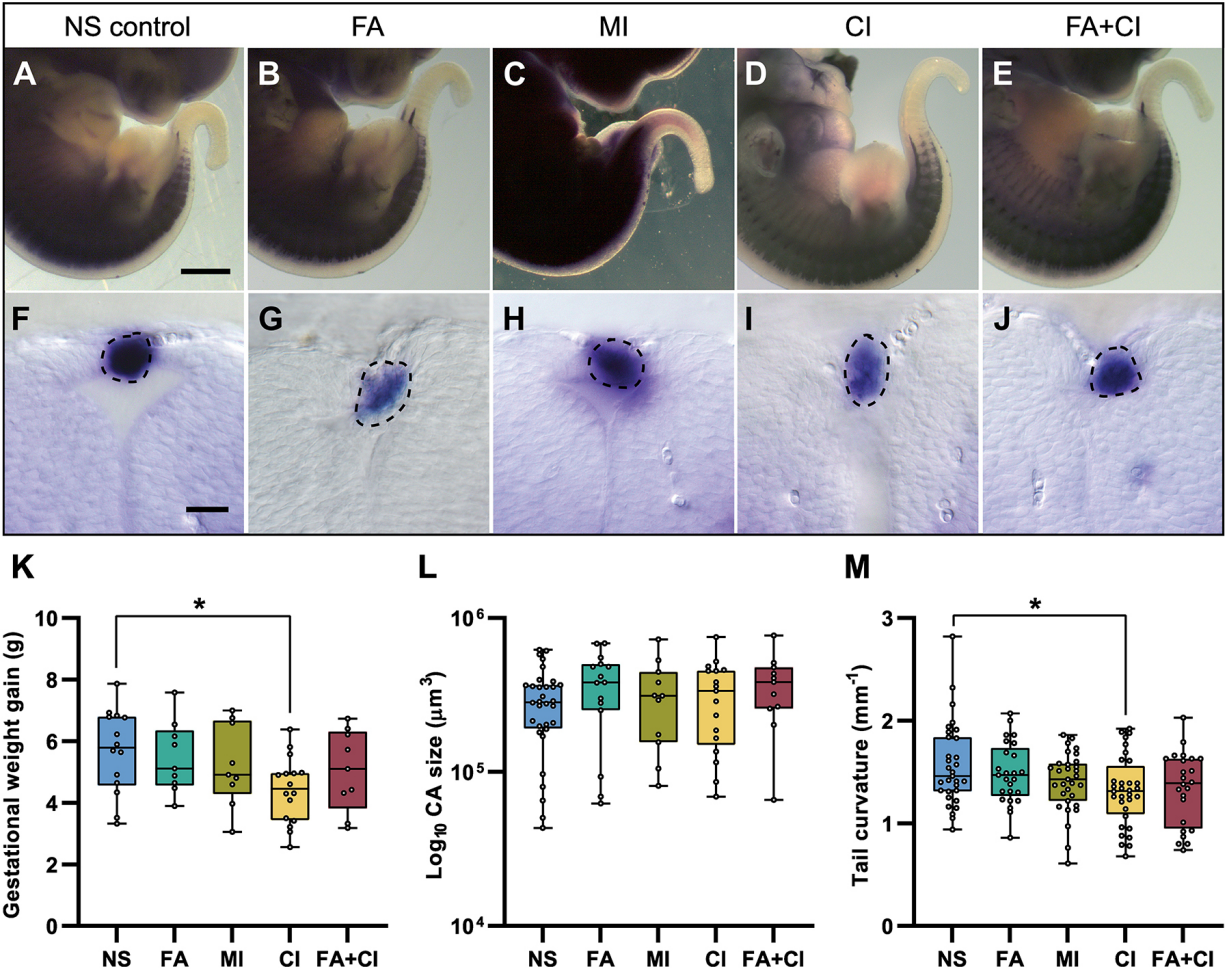
**Maternal supplementation with folic acid or inositol does not reduce the incidence of caudal dorsal fusion failure detected in *Vangl2^+/Lp^* embryos.** (A-E) Magnification of the caudal area of E12.5 *Vangl2^+/Lp^* embryos after *in situ* hybridisation with *Sox10*: non-supplemented (NS) control embryo (A); embryos from mothers exposed to folic acid (FA) (B), *myo-*inositol (MI) (C), D*-chiro*-inositol (CI) (D) or to a combination of FA and CI (FA+CI) (E). (F-J) Transverse sections through CAs located between somites 30 and 33, representative of their similarity in size under the different supplementation conditions. The dashed lines border the CAs. Images are representative of *n*=32 NS, *n*=15 FA, *n*=11 MI, *n*=17 CI and *n*=11 FA+CI embryos. Scale bars: 1000 μm (A-E); 25 μm (F-J). (K) Weight gain of female mice under the different supplementation conditions after 12.5 days of gestation. (L,M) Size of the CAs (L) and tail curvature (M) of the *Vangl2^+/Lp^* embryos under the different supplementation conditions. Boxes show the 25-75th percentiles, whiskers show the minimum and maximum, and the median is marked with a line. Ordinary one-way ANOVA followed by Dunnett's multiple comparisons test was used for statistical analysis and only significant differences are indicated. *P<0.05.

We first evaluated the safety of the supplements on the progress of the pregnancy and embryo health according to the following parameters: maternal weight gain, litter size, number of live embryos, embryo genotype ratio, embryo crown-rump length and embryonic macroscopic malformations. Although most of these parameters did not differ during gestation under any of the dietary supplementation conditions (Table S3), the CI-supplemented diet resulted in less weight gain compared to that in NS females during the 12.5 gestational days of the study (4.4±1.1 g versus 5.7±1.4 g, respectively; *P*=0.0285; Fig. 7K). None of the supplementations studied were effective in preventing occasional open NTDs associated with this mutation, such as open spina bifida or exencephaly (Table S3).

With regards to the Mendelian distribution of embryonic genotypes, an altered distribution was observed in some of the groups, although none of them were statistically significant (Table S3). Under FA supplementation, there was an altered distribution in favour of the *Vangl2^+/+^* genotype over the *Vangl2^Lp/Lp^* genotype (*P*=0.185). This trend corroborated similar observations previously reported by other groups (Nakouzi and Nadeau, 2014). Under MI supplementation, there was a clear, but not significant (*P*=0.2074), decrease of heterozygotes in the litters, resulting in an altered Mendelian ratio similar to 1:1:1. Litters from dams supplemented with CI tended to have considerably fewer *Vangl2^+/+^* embryos, as 50% (8/16) of CI litters had no *Vangl2^+/+^* embryos, compared to only 14% (2/14) of NS litters (*P*=0.2695). This absence of *Vangl2^+/+^* embryos in such a large number of litters occurred exclusively with CI and not with any other supplementation groups.

After carrying out the assessment protocol (supplementary Materials and Methods) that we developed based on *Sox10 in situ* hybridisation in *Vangl2^+/Lp^* embryos exposed to the different supplementation conditions, no differences were detected in any of the designed parameters that may indicate an improvement of the phenotype (Fig. 7A-J,L; Tables S1 and S2). The only change observed was in tail curvature, as this measurement was significantly reduced in embryos exposed to CI compared to NS embryos (1.32±0.34 mm^−1^, *n*=32, versus 1.56±0.41 mm^−1^, *n*=31; *P*=0.0164; Fig. 7M; Table S1). The FA+CI group also showed a trend, although not significant (*P*=0.056), towards a reduction in tail curvature.

## DISCUSSION

The aetiology of NTDs has not yet been fully elucidated, but numerous genetic and environmental risk factors have been described (Finnell et al., 2021; Wolujewicz et al., 2021). The presentation of NTDs varies mainly according to the location of the lesion. In this study, we present a mouse model that has three types of NTDs: craniorachischisis, open spina bifida and caudal closed NTDs. Although the first two phenotypes had been already characterised in *loop-tail* mutants (Kibar et al., 2001; López-Escobar et al., 2018; Murdoch et al., 2001), closed NTDs, by their very nature, have not been described before. This makes *Vangl2^+/Lp^* a mouse model of caudal closed NTDs, for which, here, we describe its potential origin, which will facilitate future studies of this disease. As in humans, we detected a great heterogeneity in the severity of this closed NTD in our murine model, therefore we developed a systematic protocol for assessing the severity of caudal NT fusion failure (supplementary Materials and Methods). We anticipate that this protocol could be a useful tool to standardise the study in other mutants with closed NTDs to assess genetic interactions and to design new disease-prevention strategies.

Our previous studies have shown that the neuroepithelium of the posterior neuropore of *Vangl2^+/Lp^* embryos (Wnt-PCP pathway mutants) exhibits a series of cytoskeletal alterations that affect cell morphology and polarity (López-Escobar et al., 2018). In the course of this detailed study of caudal closure of the NT in *Vangl2^+/Lp^* embryos at earlier stages, the phenotype of dorsal fusion failure with the consequent appearance of CAs was not observed. As shown here, these malformations observed in *Vangl2^+/Lp^* embryos resulted in an absence of dorsal fusion of the neural folds in the most caudal part of the primary neurulation, specifically in the transition zone between primary and secondary neurulation (Fig. 4C). This failure appeared to be sealed by a group of cells from the neural crest, which could facilitate the sealing of the defect by the surface ectoderm, phenocopying the closed NTDs in humans. Interestingly, as is the case with the craniorachischisis phenotype in homozygosity (Kibar et al., 2001; Murdoch et al., 2001), the failure of NT dorsal fusion is fully penetrant in *Vangl2^+/Lp^* embryos, and we believe that only in the most severe cases, where the affected area is too large, an open spina bifida would develop.

### Progression of the close NTD phenotype in *Vangl2^+/Lp^* embryos

Arguably, abnormalities solely due to failures in secondary neurulation in mice would by definition generate closed NTDs. Thus, observing a failure of neural fold fusion, even when covered by epithelium, as we see in our model, would suggest that it is due to a process affecting primary neurulation or its progression to secondary neurulation. This is a very interesting point that could be the key to why, in our model, CAs disappear over time. We consider two possible reasons for this finding. One may lie in the different processes involved in the transition from primary to secondary neurulation in mice and humans. Although not fully defined, it appears that in humans, the onset of secondary neurulation and the transition from primary to secondary neurulation resembles more closely the process in chick embryos than in mice embryos. In chick embryos, in which this process is well defined, at the level of the posterior neuropore, there is a zone of overlap between the end of primary (dorsal) neurulation and the beginning of secondary (ventral) neurulation (Catala, 2021; Dady et al., 2014). In mouse embryos, the lumen of the primary neurulation is continued with the onset of secondary neurulation (Shum and Copp, 1996). In our murine model, the CAs seemed to appear at sites where there was a failure of the final fusion of the primary neurulation, and we hypothesise that, as the formation of the secondary neurulation lumen continues to progress, a disintegration or incorporation of the CA cells into the adjacent tissue may occur. A transition process from primary to secondary neurulation different from that in mice could affect the disappearance of CAs, which may remain anchored at the site of formation, growing and differentiating into the different tissues that form the lipoma, as observed in humans. Interestingly, chickens present a higher incidence of caudal NTDs than mice under various experimental conditions (Hughes and Freeman, 1974; Schoenwolf, 1979). The way in which CAs disappear in mouse embryos could either be by disintegration into single cells in the lumen and death, or by undergoing a process of transition from the mesenchyme to epithelium and being incorporated into the adjacent NT *Sox10^+^* population. The latter option would be interesting to investigate further, as occasionally *Sox10* expression appeared to be increased at late stages in the *Sox10^+^* NT cell population in *Vangl2^+/Lp^* embryos (Fig. 5K,L). The possibility of mesenchymal-to-epithelial transition, followed by incorporation into the adjacent epithelium, would also explain the disappearance of CAs located above a fused NT, resulting in their incorporation into either the neuroectoderm or surface ectoderm.

An alternative explanation would be that in humans, the development and clearance of CAs may also take place and, like in mice, it is when a combination of several risk factors occurs that the phenotype may become more severe and prevail. This idea would be in line with the multifactorial threshold model of NTDs in humans (Harris and Juriloff, 2007), in which the defect occurs as a result of an additive contribution of several risk factors, each of which is individually insufficient to disrupt neurulation. To shed light on this matter, it might be relevant to ascertain whether *Vangl2^+/Lp^/Grhl3^+/Ct^* double mutants show CAs at later stages than E14.5.

Here, we present how the presence of NT fusion failure could cause a group of NCCs undergoing EMT to accidentally change their fate and become trapped in the ectopic opening. Moreover, the mesenchymal nature of CA cells (*Sox10^+^*, Fig. 3F; Fig. S2E,F) and the expression of adipocyte differentiation markers (PDGFRα, Fig. 3D; Fig. S1K,L; Hepler et al., 2017), suggests that the dorsal defect we observed in *Vangl2^+/Lp^* embryos may well resemble a lipomyelomeningocele primordium in humans. In regard to this, a previous study of patients with NTDs reported a higher incidence of *VANGL2* mutations in cases with closed spinal NTDs (2.5%, compared to 0.5% in open spina bifida); these cases of closed spinal NTDs with *VANGL2* mutations also showed a higher prevalence of lipoma (Kibar et al., 2011).

### Additive contribution of mutations of genes associated to NTDs in the development of NT dorsal fusion failure

The protocol we developed for assessing the severity of dorsal damage associated with the *Vangl2* gene mutation (supplementary Materials and Methods) was validated with the phenotypes resulting from combination of *Vangl2* mutants with *Damm1* and *Grhl3* mutants. The *Vangl2^+/Lp^/Daam1^+/gt^* double mutants showed a significant increase in the size of the CAs, suggesting a more severe phenotype (Fig. 6; Table S1). The area most affected by this double mutation was located in the 30-33s region, precisely the zone where *Vangl2^+/Lp^* embryos showed the highest incidence of CAs (Fig. 6M,O,P). These results suggest that the region most sensitive to mutations in the Wnt-PCP pathway may be located at this specific anteroposterior level of the embryo. We previously observed an incidence of 21% of open spina bifida in these double mutants (López-Escobar et al., 2018). Thus, we hypothesise that the double mutation of members of the Wnt-PCP pathway may lead to a severe impairment of the morphology of the caudal neural plate, giving rise to a single phenotype that, in the most severe cases, results in open spina bifida and, in the less severe cases, in closed caudal NTD. As 100% of the embryos examined had either open or closed spina bifida, we concluded that this phenotype of caudal NTD is 100% penetrant. For future work in the field of caudal NTDs, it would be interesting if, when describing a phenotype of open spina bifida in mouse models, the precise anteroposterior location where the damage begins would be indicated, as this could facilitate its classification and translationality to humans.

*Vangl2/Grhl3* double-mutant embryos showed the most severe closed caudal NTDs as evidenced by the presence of higher numbers of and larger CAs, which were mainly found in the most anterior region (≤29s). In addition to close caudal NTDs, we observed in our litters that 1/28 (1.8%) *Vangl2^+/Lp^/Grhl3^+/Ct^* embryos developed open spina bifida, whereas 4/28 (14.3%) presented exencephaly. The *Grhl3* and the *Vangl2* mutants share certain features of interest. On the one hand, the two described mechanisms that are altered in these mutants affect a mechanical process, the failure of which prevents dorsal fusion of the NT. *Grhl3* mutants show reduced proliferation in the hindgut and notochord, which, together with the normal proliferation of the neuroepithelium, results in an increased curvature of the caudal region, which in turn impairs NT closure (Gustavsson et al., 2007). *Vangl2*, as part of the Wnt-PCP pathway, modulates the contraction of the apical cytoskeleton of the caudal neuroectoderm, the failure of which prevents proper folding of the tissue for subsequent fusion (López-Escobar et al., 2018). The combined disturbance of these two independent mechanical processes could be the cause of the greater weakness in the dorsal fusion of the NT in the caudal area. Moreover, these two mutants also share the characteristic of being resistant to FA supplementation (Nakouzi and Nadeau, 2014; Seller, 1994), as is the case with closed NTDs in humans (De Wals et al., 2008; Forrester and Merz, 2004; McNeely and Howes, 2004). Thus, we hypothesise that there are FA-resistant caudal NTDs, the phenotype of which varies in severity from open spina bifida to a milder phenotype of lipomyelomeningocele with no physical problems, and that this heterogeneity may be due to the combination of risk factors present during neurulation.

### Screening of maternal supplements for the prevention of NTDs associated with the Wnt-PCP pathway

Supplementation of the maternal diet with FA has been well documented to reduce the incidence of NTDs in humans by 30-40% in the general population and by 70% for maternal supplementation with high levels of FA following a previous pregnancy with NTDs (De Wals et al., 2007; MRC Vitamin Study Research Group, 1991). FA has been shown to modify epithelial cell shape during morphogenesis by facilitating Rho kinase-dependent apical constriction (Martin et al., 2019). Knowing that *Vangl2^+/Lp^* embryos exhibit apical constriction problems in the caudal neuroectoderm (López-Escobar et al., 2018), we hypothesised that FA supplementation could reverse this phenotype. However, our work showed that in the *loop-tail* mouse model, maternal FA did not reduce the incidence of open spina bifida or closed NTDs (Table S3), neither did it reduce the incidence of craniorachischisis here and in a previous study (Nakouzi and Nadeau, 2014). In humans, FA also has no preventive effect on lipomyelomeningocele (De Wals et al., 2008; Forrester and Merz, 2004; McNeely and Howes, 2004). These observations, together with the phenotypic features described in this work, suggest that *Vangl2^+/Lp^* mutants may be a good model of the closed NTD lipomyelomeningocele.

Given that some NTDs are FA-resistant, research in this field has been directed towards the identification of other nutritional factors that may contribute to the prevention of NTDs, such as inositol (Greene et al., 2016). Inositol and its derivatives are involved in the organisation and dynamics of the cell membrane and can be coupled to the underlying F-actin cytoskeleton via linker proteins (Logan and Mandato, 2006). Given that F-actin cytoskeleton is impaired in *Vangl2^+/Lp^* embryos (López-Escobar et al., 2018), we sought to establish whether inositol supplementation could have a greater preventive effect than FA. However, our experiments showed that inositol (MI or CI), either alone or in combination with FA, had no effect on reducing the occurrence or severity of the caudal NT fusion failure in *Vangl2^+/Lp^* embryos (Fig. 7; Table S3). Similar to our results, other studies have shown that NTDs associated to mutations in the genes *Mekk4* (*Map3k4*), *Grhl3* and *Fkbp8* were resistant to both FA and inositol supplementation (Chi et al., 2005; Ting et al., 2003; Wong et al., 2008). *Zic3* mutants also showed resistance to folinic acid, zinc and inositol (Franke et al., 2003). To date, only the *curly tail* mutant has been described as a FA-resistant NTD mouse model that responds to maternal inositol supplementation (Greene and Copp, 1997).

### Maternal dietary supplementation alters Mendelian distribution in *loop-tail* offspring

Similar to our results, other groups have also observed in *loop-tail* and other mouse NTD models that maternal dietary FA supplementation does not reduce the incidence or severity of NTDs, but causes an alteration in the balance of the Mendelian distribution of the three genotypes without an increase in apparent embryonic death or reduction in litter size (Gray et al., 2010; Marean et al., 2011; Nakouzi and Nadeau, 2014). During the course of our work, we observed the three scenarios: FA supplementation appeared to favour the *Vangl2^+/+^* genotype over the *Vangl2^Lp/Lp^* genotype; MI reduced the number of *Vangl2^+/^*^Lp^ embryos, matching their number to that of the other two genotypes; and with CI supplementation, surprisingly, it was the *Vangl2^+/+^* genotype that was mostly depleted, favouring the mutated *Vangl2^+/Lp^* and *Vangl2^Lp/Lp^* genotypes. Embryonic death of homozygous embryos for a mutation has generally been justified as a product of the elimination of the weaker genotype. However, this cannot be the answer to the loss of heterozygous and wild-type embryos, mainly in the absence of embryonic loss. The most notable exceptions to random segregation are the infrequent examples of transmission ratio distortion found in nature, which have been described from fungi to humans (reviewed in Nadeau, 2017). These uncommon events suggest that fertilisation is genetically biased towards certain gametes on the basis of their genetic content. Along with *loop-tail* (Nakouzi and Nadeau, 2014), four other NTD mutants susceptible to transmission ratio distortion under the effects of maternal dietary supplementation with FA have been described (Gray et al., 2010; Marean et al., 2011; Nakouzi and Nadeau, 2014). Not only that, but our results show that *loop-tail* exhibits different Mendelian distributions according to maternal supplementation. Although with our data, we cannot explain this observation, we believe it is interesting to report, so that together with the work of other researchers, the effect of maternal diet on possible alterations of the Mendelian distribution can be elucidated, to which the *loop-tail* mutant seems to be especially sensitive.

Overall, our work suggests that loss of *Vangl2* affects molecular pathways that are resistant to FA- and inositol-mediated rescue effects, which, together with the varying degree of severity of caudal NTDs observed in *loop-tail* embryos, makes *VANGL2* an important candidate gene for untreatable caudal NTDs in human patients. Therefore, as research in this field progresses, it may be possible to clarify the aetiology and susceptible pathways of prevention protocols that could be taken into account when classifying the typology of NTDs, thereby helping to develop more personalised prevention strategies.

## MATERIALS AND METHODS

### Mice

The *loop-tail* (*Vangl2^Lp^*) inbred strain carrying the *Vangl2* mutation was originally obtained from Jackson Laboratories, and it was maintained in a C3H background. To produce *Vangl2^+/Lp^* and *Vangl2^Lp/Lp^* embryos, *Vangl2^+/Lp^* females were crossed with *Vangl2^+/Lp^* males. The *Daam1* gene trap mutant mice were obtained from BayGenomics (RRT390) (Li et al., 2011) and were maintained on a C57 background. To produce double-heterozygote *Vangl2^+/Lp^/Daam1^+/gt^* mice, *Daam1^+/gt^* mice were crossed with *Vangl2^+/Lp^* mice. The *Ghrl3* mutant mice were kindly provided by Prof. Andrew J. Copp (UCL Great Ormond Street Institute of Child Health) and were crossed with *Vangl2^+/Lp^* mice to produce *Vangl2^+/Lp^/Ghrl3^+/Ct^* embryos. After overnight mating, dams were checked for vaginal plugs and the day on which a copulation plug was found was designated as E0.5. The mice were maintained on a 12 h/12 h light/dark cycle (lights on from 08:00 to 20:00), and mice had *ad libitum* access to food and water. All procedures involving experimental animals were performed in compliance with local, national and European animal welfare laws, guidelines and policies.

### FA, MI and CI supplementation

For maternal FA supplementation, *Vangl2^+/Lp^* females and *Vangl2^+/Lp^* males were weaned at 4 weeks of age and thereafter maintained on either a standard diet containing 2 ppm FA (TD.190813, Teklad Global 14% Protein Rodent Maintenance Diet, 2014, Italy) or a supplemented diet containing 10 ppm FA (TD.190813, Teklad Global 14% Protein, 2014, 10 ppm FA, US) for at least 2 weeks prior to mating. Diets were identical except for the FA increase in the supplemented diet. Upon discovery of a plug, females were kept on the same diet until they were sacrificed. For inositol treatment, either MI (AppliChem, A17160100) or CI (Santa Cruz Biotechnology, sc-221469) diluted in sterile distilled water were administered to *Vangl2^+/Lp^* pregnant dams from E1.5 to E11.5. The supplemented water was prepared in opaque bottles and changed every two days to protect inositol from light exposure. The standard water was sterile distilled water without any solute. The concentration used for both MI and CI was 800 µg/g of weight/day (Cogram et al., 2002). The average weight of a mouse was 25 g and it drank an average of 5 ml of water/day. Timed pregnancies were generated by crossing 2- to 6-month-old females with males overnight. The mean female age, shown as months±s.e.m., was the following per group: NS, 3.7±0.3 (*n*=14); FA, 3.5±0.5 (*n*=9); MI, 4.7±2.7 (*n*=9); CI, 5.0±0.3 (*n*=16); and FA+CI, 4.0±0.5 (*n*=9).

### Embryo collection, genotyping and crown-rump length measurement

Pregnant females were sacrificed at E11.5-E14.5 and the total number of implants, classified as viable embryos, malformed embryos or resorptions, was recorded. Yolk sacs were used for *Vangl2* and *Daam1* embryos genotyping as described previously (Li et al., 2011; Stanier et al., 1995). All embryos were analysed macroscopically and photographed with a stereomicroscope (SteREO Discovery V8, Zeiss) coupled to an AxioCam Erc8 camera (Zeiss). The presence or absence of the characteristic curly-tail phenotype of *Vangl2^+/Lp^* embryos was examined, as well as the appearance of other external malformations (open spina bifida, exencephaly or craniorachischisis). The crown-rump length of the supplemented and control embryos was measured following a standard protocol (García-Peláez et al., 2010).

### Tissue preservation

For *in situ* hybridisation, embryos were fixed in 4% paraformaldehyde (PFA), for 24-48 h at 4°C, washed in PBS, serially dehydrated to 100% methanol, and stored at −20°C until further use. For immunohistochemistry and HABP and phalloidin staining, embryos were fixed in 4% PFA at 4°C for 12 h, washed in PBS, submerged in 7.5% sucrose in PBS until they sank to the bottom, and then stored in 15% sucrose solution for 12 h. The embryos were then embedded in gelatine (15% sucrose and 7.5% gelatine in PBS) and positioned so that the area just below the hind limbs (region between somites 25 and 30) was oriented to obtain good cross-sections of the area. Embryos were then frozen by immersion in 2-methyl-butane for approximately 1 min and stored at −80°C until cryosectioned at 10 or 20-µm-thick sections using a Leica CM1950 cryostat. For histological staining, embryos were fixed for 2-4 h in 4% PFA, dehydrated serially in ethanol, followed by embedding in paraffin wax and transverse sectioning using a microtome (7 μm thick). The sections were stained with Ehrlich Haematoxylin and Eosin and imaged on an Olympus BX-61 microscope using 20× and 40× objectives.

### Immunofluorescence

After removal of the gelatine from the frozen cross sections, these were incubated in blocking solution (PBS, 1% bovine serum albumin and 0.1% Triton X-100) for 20 min. They were then incubated with the corresponding primary antibody diluted in the blocking solution overnight at 4°C. After washing with PBS, they were incubated with the secondary antibody diluted in blocking solution at room temperature for 1 h (antibody details are shown in Table S4). After washing with PBS, the sections were mounted with Hydromount (National Diagnostics, HS-106). To localise F-actin, sections were incubated with phalloidin-isothiocyanate tetramethylrhodamine B (Sigma-Aldrich, P1951) diluted 1:500 in PBS and 0.1% Triton X-100 (PBST) for 1 h at room temperature. In the same way, HA was detected using biotin-conjugated HABP (1:100; Calbiochem, 385911). After mounting, 10-μm-thick sections were imaged on the Olympus BX61 microscope, whereas 20-μm-thick sections were imaged on a confocal microscope (Leica TCS-SP2-AOBS or Nikon A1R^+^ confocal microscopes).

### Whole-mount *in situ* hybridisation

Whole-mount *in situ* hybridisation was carried out using sense and antisense digoxigenin-labelled RNA probes prepared using a digoxigenin RNA labelling kit (Roche, 11175025910) according to the manufacturer's instructions. As described previously (Ybot-González et al., 2005), mouse embryos were analysed with the probe for *Sox10* (Britsch et al., 2001). Whole embryos were photographed on a stereomicroscope and were embedded in a gelatine-sucrose-albumin and glutaraldehyde solution. Vibratome transverse sections of the caudal NT (50-μm-thick) of the embryos were photographed at 20× and 40× on the Olympus BX-61 microscope using the same imaging parameters for all samples. CAs were identified by the presence of dorsal *Sox10* labelling.

### Assessment protocol for CA-associated dorsal damage

This study was performed on E11-E13.5 embryos after being exposed to whole-embryo *in situ* hybridisation using the *Sox10* probe. We developed a protocol to assess the severity of the caudal NT fusion failure in *Vangl2^+/Lp^* embryos using the CAs as a readout based on six parameters; more specific instructions on consistently assaying closed NTDs as found in *Vangl2^+/Lp^* embryos can be found in the supplementary Materials and Methods. First, whole embryos were analysed and photographed at 8× magnification with a stereomicroscope (SteReo CL 1500 ECO). These images showed the number of CAs per embryo as well as their location on the anteroposterior axis with reference to the corresponding somite. This information was used for the parameters: (1) incidence, (2) number of regions affected by NT fusion failure and (3) location of NT fusion failure. Transverse sections of the different CAs along the body of the embryo were photographed under an Olympus BX-61 microscope at 40×. These images were then analysed to measure the size of each CA (using the ImageJ ‘polygon’ tool) and to visually determine whether each CA was associated with a closed or open NT. The area taken into consideration was the entire CA, as observed morphologically, and was not limited to the area marked with *Sox10*. Subsequently, the extent of damage in the dorsal NT was calculated by summing up the areas of all the CAs found in an embryo, and multiplying this number by the thickness of the sections (50 µm) in order to obtain the total CA size in µm^3^. This information was used for the parameters: (4) NT area affected by dorsal fusion failure and (5) late NT closure progression. For parameters 2 (number of regions) and 4 (NT area affected by dorsal fusion failure), 0 was considered when an embryo did not present any CAs in a given zone. Finally, from the images of whole embryos, the curvature (κ) of the caudal region was obtained by measuring the radius (*r*) of a circumference that was drawn (using ImageJ software) following the curvature of the tail of *Vangl2^+/Lp^* embryos and applying the formula κ=1/*r*. This information was used for parameter 6 (tail curvature).

### Statistical data analysis

GraphPad Prism 9 was used to plot the data and to perform statistical analyses. Ordinary one-way ANOVA followed by Tukey's multiple comparisons test was used for comparison of CA number and size in *Vangl2^+/Lp^* embryos (Fig. 4H,I). Ordinary one-way ANOVA followed by Dunnett's multiple comparisons test was used for comparison of the tail curvature in double mutants against *Vangl2^+/Lp^* embryos (Fig. 6N) and the different supplementations against non-supplemented embryos (Fig. 7K-M; Table S3). Unpaired two-tailed *t*-test was used for the comparisons of total CA number and size between *Vangl2^+/Lp^* embryos and double mutants (Fig. 6O-R). Two-way ANOVA followed by Šídák's multiple comparisons test was used for the comparisons of CA number and size per zones between *Vangl2^+/Lp^* embryos and double mutants (Fig. 6O-R). χ^2^ test was used for the comparison of the CA distribution along defined anteroposterior zones (Fig. 6M) and the Mendelian distribution of embryo genotypes with supplementations (Table S3). All data are presented in the text and tables as mean±s.d. and *P*<0.05 was considered statistically significant.

## Supplementary Material

10.1242/dmm.050175_sup1 Supplementary information
