# Supplementary material for: The loop-tail mouse model displays open and closed caudal neural tube defects
Source: Dis Model Mech. 2023 Aug 29;16(8):dmm050175. doi: 10.1242/dmm.050175 (PMC10481946; doi:10.1242/dmm.050175)
Supplement: Supplementary information [file dmm-16-050175-s1.pdf]

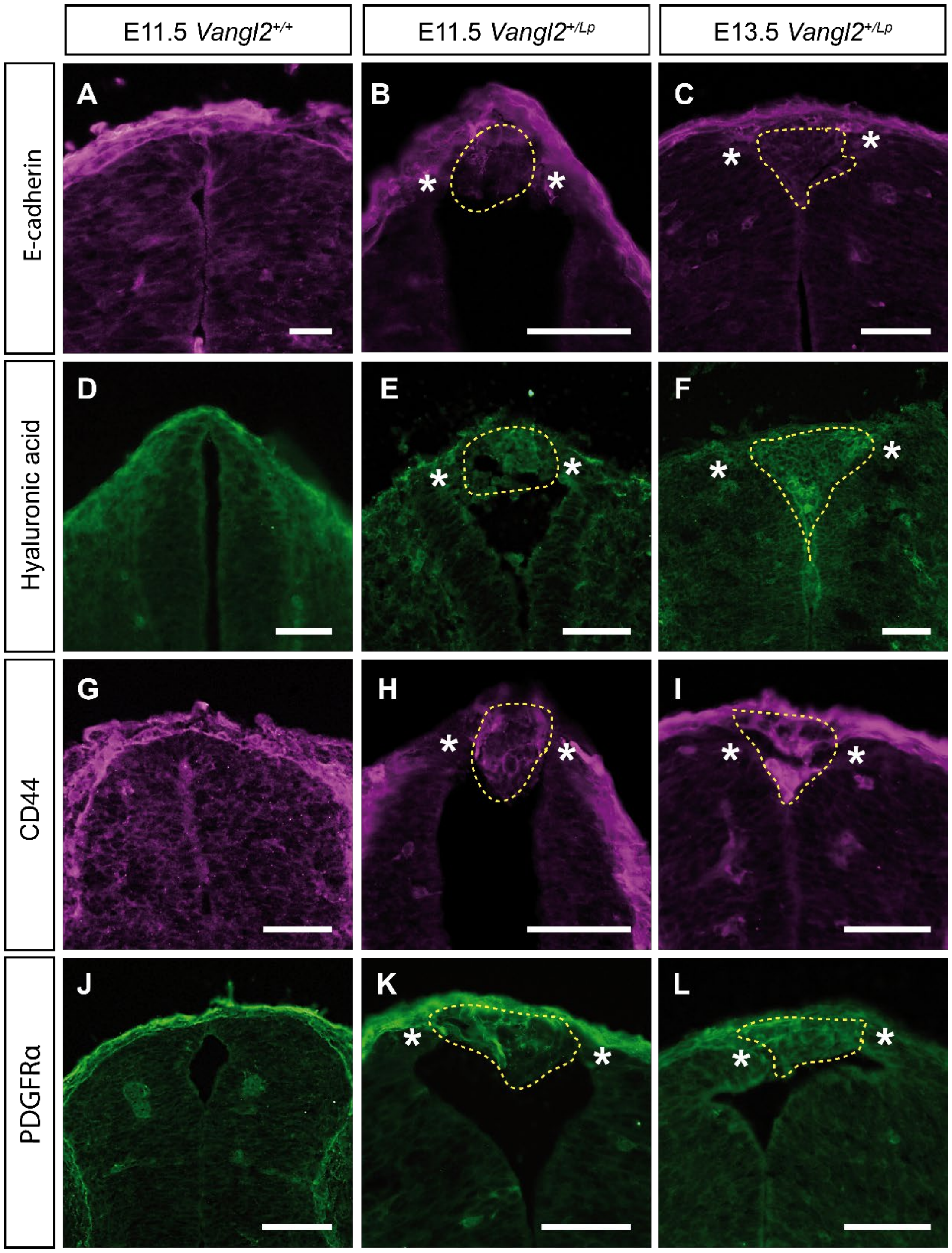

Fig. S1. Insights into the molecular profile of the cellular aggregate (CA) using wound healing machinery markers. Transverse sections of CAs from E11.5 *Vangl2*$^{+/+}$ (left panels), E11.5 *Vangl2*$^{+/Lp}$ (middle panels), and E13.5 *Vangl2*$^{+/Lp}$ embryos (right panels) immunolabelled for: (A-C) E-cadherin, (D-F) hyaluronic acid binding protein, HABP, (G-I) CD44, and (J-L) platelet-derived growth factor receptor α, PDGFRα. The yellow dotted lines delimit the CAs. Asterisks (*) mark the dorsal tip of the opposing neural folds, highlighting the failure of NT dorsal fusion of *Vangl2*$^{+/Lp}$ embryos at these post-fusion stages. At least n=3 embryos per marker were analysed. Scale bars: 50 µm.

Disease Models & Mechanisms • Supplementary information

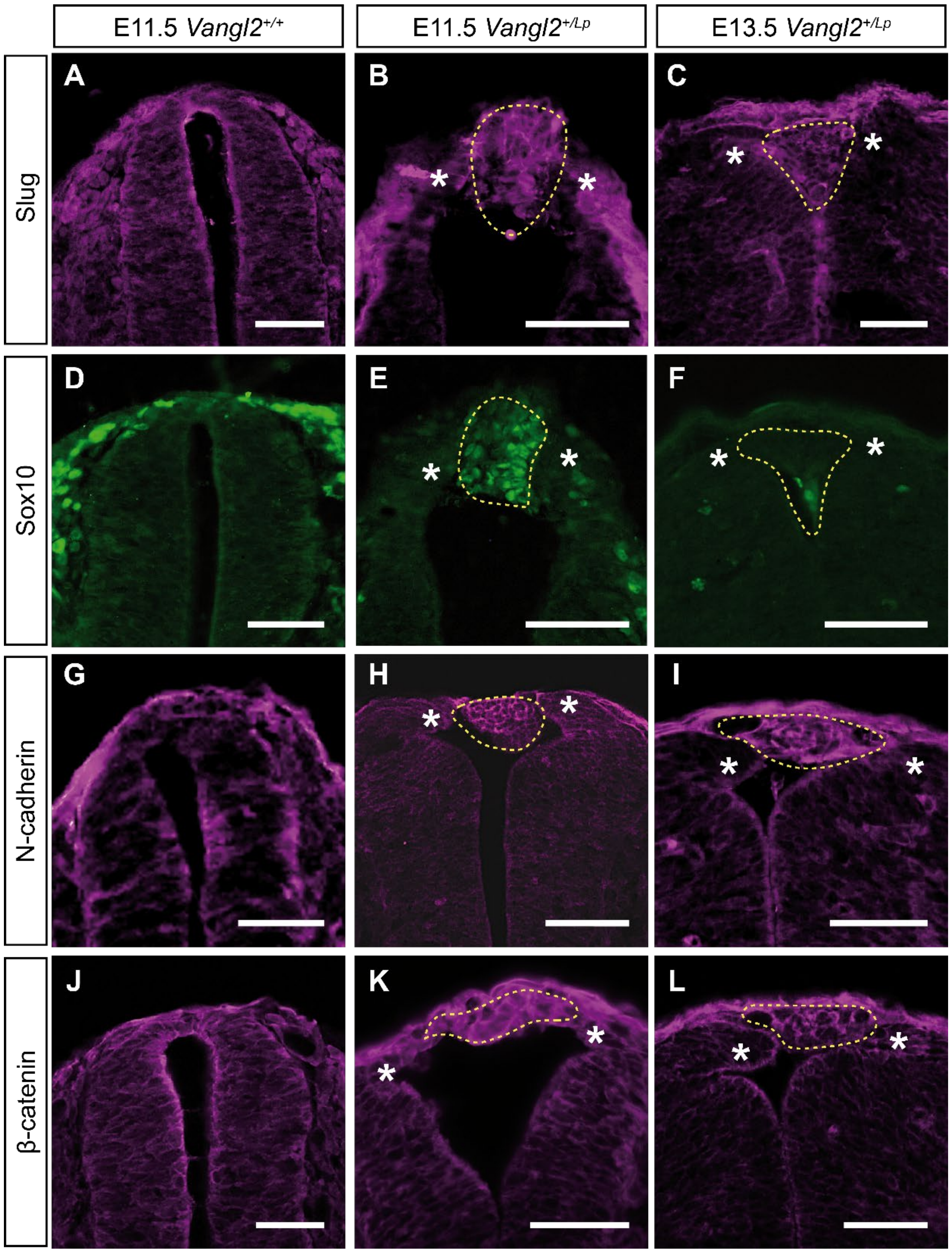

Fig. S2. Insights into the molecular profile of the cellular aggregate (CA) using neural crest cells and cadherin switch markers. Transverse sections of CAs from E11.5 *Vangl2*$^{+/+}$ (left panels), E11.5 *Vangl2*$^{+/Lp}$ (middle panels), and E13.5 *Vangl2*$^{+/Lp}$ embryos (right panels) immunolabelled for: (A-C) Slug, (D-F) Sox-10, (G-I) N-cadherin, and (J-L) β-catenin. The yellow dotted lines delimit the CAs. Asterisks (*) mark the dorsal tip of the opposing neural folds, highlighting the failure of NT dorsal fusion of *Vangl2*$^{+/Lp}$ embryos at these post-fusion stages. At least *n*=3 embryos per marker were analysed. Scale bars: 50 µm.

Disease Models & Mechanisms • Supplementary information

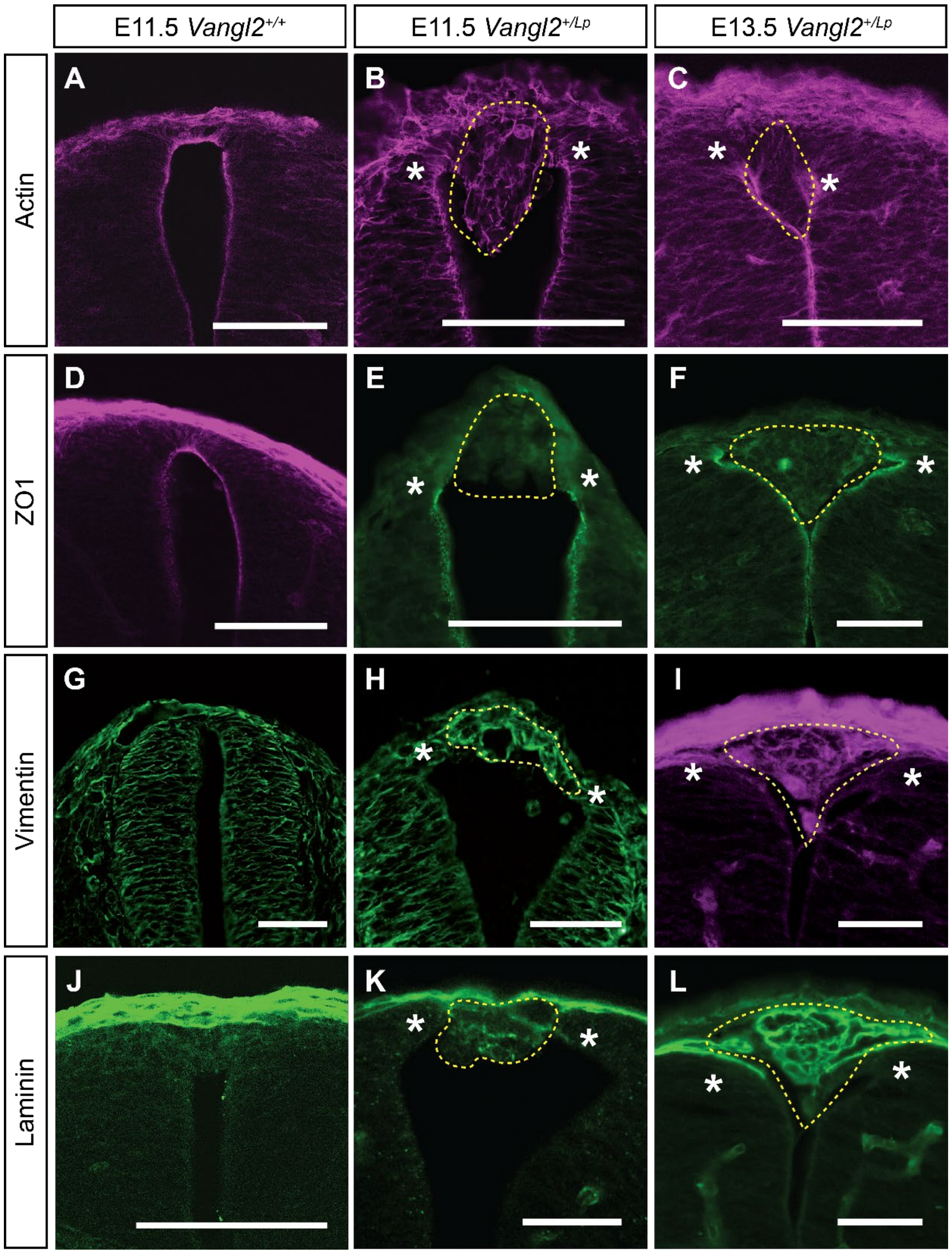

Fig. S3. Insights into the molecular profile of the cellular aggregate (CA) using cytoskeleton and polarity markers. Transverse sections of CAs from E11.5 *Vangl2*$^{+/+}$ (left panels), E11.5 *Vangl2*$^{+/Lp}$ (middle panels), and E13.5 *Vangl2*$^{+/Lp}$ embryos (right panels) immunolabelled for: (A-C) phalloidin, used to visualise actin distribution, (D-F) ZO1, (G-I) vimentin, and (J-L) laminin. The yellow dotted lines delimit the CAs. Asterisks (*) mark the dorsal tip of the opposing neural folds, highlighting the failure of NT dorsal fusion of *Vangl2*$^{+/Lp}$ embryos at these post-fusion stages. At least *n*=3 embryos per marker were analysed. Scale bars: 50 µm.

Disease Models & Mechanisms • Supplementary information

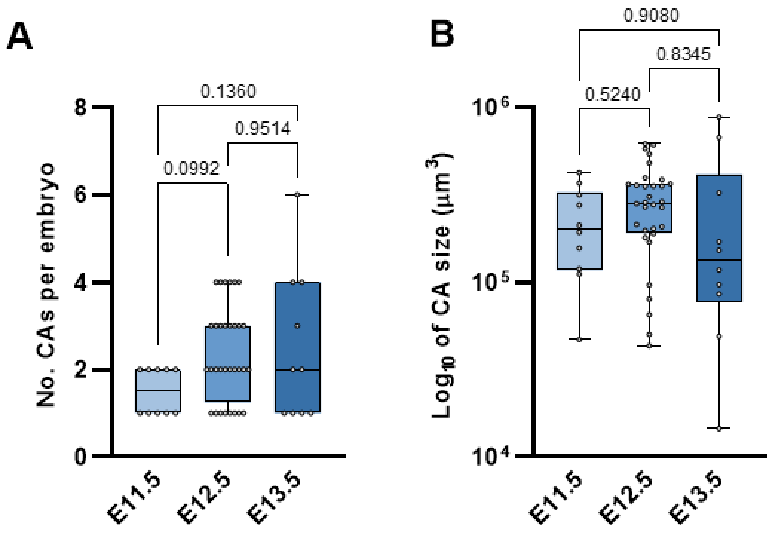

Fig. S4. Characteristics of the cellular aggregates (CAs) of *Vangl2*$^{+/Lp}$ embryos at E11.5-13.5. (A) Number and (B) size of the CAs found in E11.5 (*n*=10), E12.5 (*n*=32) and 13.5 (*n*=10) *Vangl2*$^{+/Lp}$ embryos. One-way ANOVA followed by Tukey's multiple comparisons test was used for statistical analysis; exact *P*-values are indicated.

Disease Models & Mechanisms • Supplementary information

Table S1. General characteristics of the phenotype of the embryos analysed in the study using the CAs as a readout of dorsal NT closure failure.

Genotype	Condition	Incidence (%)	No. CAs per embryo	Embryos with a given number of CAs (%)							CA size ($\times 10^5$ µm^3)	CAs associated to closed NT (%)	Tail curvature (mm^{-1})
				1-CA	2-CA	3-CA	4-CA	5-CA	6-CA	7-CA			
Embryos E11.5-13.5													***Embryos E12.5***
Vangl2$^{+/Lp}$	NS	100%<br>(52)	2.2±1.2<br>(116)	32.7%<br>(17/52)	32.7%<br>(17/52)	17.3%<br>(9/52)	15.4%<br>(8/52)	0%	1.9%<br>(1/52)	0%	2.74±1.83<br>(52)	23.3%<br>(27/116)	1.56±0.41<br>(31)
Embryos E12.5-13.5													***Embryos E12.5***
Vangl2$^{+/Lp}$	NS	100%<br>(42)	2.4±1.2<br>(101)	28.6%<br>(12/42)	28.6%<br>(12/42)	21.4%<br>(9/42)	19.0%<br>(8/42)	0%	2.4%<br>(1/42)	0%	2.87±1.94<br>(42)	26.7%<br>(27/101)	1.56±0.41<br>(31)
Vangl2$^{+/Lp}$/<br>*Daam1*$^{+/gt}$	NS	100%<br>(12)	2.2±1.1<br>(27)	33.3%<br>(4/12)	25.0%<br>(3/12)	25.0%<br>(3/12)	16.7%<br>(2/12)	0%	0%	0%	**5.78±3.17*****<br>(12)	33.3%<br>(9/27)	1.32±0.39<br>(6)
Embryos E11.5-12.5													***Embryos E12.5***
Vangl2$^{+/Lp}$	NS	100%<br>(42)	2.2±1.0<br>(91)	30.9%<br>(13/42)	35.7%<br>(15/42)	19.0%<br>(8/42)	14.3%<br>(6/42)	0%	0%	0%	2.79±1.51<br>(42)	13.2%<br>(12/91)	1.56±0.41<br>(31)
Vangl2$^{+/Lp}$/<br>*Grhl3*$^{+/Ct}$	NS	100%<br>(15)	**4.1±1.4******<br>(61)	**0.0%******<br>(0/15)	**13.3%******<br>(2/15)	26.6%<br>(4/15)	20.0%<br>(3/15)	26.6%<br>(4/15)	6.7%<br>(1/15)	6.7%<br>(1/15)	**10.13±5.20******<br>(15)	16.4%<br>(10/61)	**2.13±0.44****<br>(7)
Embryos E12.5													***Embryos E12.5***
Vangl2$^{+/Lp}$	NS	100%<br>(32)	2.4±1.1<br>(76)	25.0%<br>(8/32)	31.3%<br>(10/32)	25.0%<br>(8/32)	18.8%<br>(6/32)	0%	0%	0%	2.96±1.57<br>(32)	15.8%<br>(12/76)	1.56±0.41<br>(31)
Vangl2$^{+/Lp}$	FA	100%<br>(15)	2.7±1.1<br>(40)	20.0%<br>(3/15)	20.0%<br>(3/15)	33.3%<br>(5/15)	26.7%<br>(4/15)	0%	0%	0%	3.75±2.01<br>(15)	20.0%<br>(8/40)	1.49±0.30<br>(25)
Vangl2$^{+/Lp}$	MI	100%<br>(11)	2.5±1.1<br>(28)	18.2%<br>(2/11)	36.4%<br>(4/11)	18.2%<br>(2/11)	27.3%<br>(3/11)	0%	0%	0%	3.20±1.95<br>(11)	7.1%<br>(2/28)	1.40±0.29<br>(31)
Vangl2$^{+/Lp}$	CI	100%<br>(17)	3.0±1.1<br>(51)	11.8%<br>(2/17)	17.7%<br>(3/17)	29.4%<br>(5/17)	41.2%<br>(7/17)	0%	0%	0%	3.21±1.87<br>(17)	11.8%<br>(6/51)	**1.32±0.34***<br>(32)
Vangl2$^{+/Lp}$	FA+CI	100%<br>(11)	2.8±0.9<br>(31)	9.1%<br>(1/11)	18.2%<br>(2/11)	54.5%<br>(6/11)	18.2%<br>(2/11)	0%	0%	0%	3.72±1.85<br>(11)	16.1%<br>(5/31)	1.34±0.36<br>(24)

This table represents the different measurements carried out using the CAs to assess the severity of the phenotype observed in the different experimental groups of the study: *Vangl2*$^{+/Lp}$ embryos; the double mutants *Vangl2*$^{+/Lp}$/*Daam1*$^{+/gt}$ and *Vangl2*$^{+/Lp}$/*Grhl3*$^{+/Ct}$; and *Vangl2*$^{+/Lp}$ embryos under different supplementation conditions: NS, not supplemented; FA, supplemented with folic acid; MI, supplemented with *myo*-inositol; CI, supplemented with D-*chiro*-inositol; FA+CI, supplemented with the combination of folic acid and D-*chiro*-inositol. The incidence represents the percentage of embryos with CAs, including the total number of embryos analysed in brackets. The number of CAs per embryo is shown as mean ± SD with the total number of CAs in brackets. The significant increase in the number of CAs in *Vangl2*$^{+/Lp}$/*Grhl3*$^{+/Ct}$ vs *Vangl2*$^{+/Lp}$ embryos was determined using an unpaired *t* test (P<0.0001). The number of CAs that can be found in an embryo, from 1 to 7, is represented by the percentage of embryos that present a given number of CAs, with the total number of embryos shown in brackets. The decrease in the percentage of *Vangl2*$^{+/Lp}$/*Grhl3*$^{+/Ct}$ embryos that only had 1 or 2 CAs compared to *Vangl2*$^{+/Lp}$ embryos was statistically significant as determined by Chi-square test (P<0.0001). The NT area affected by dorsal fusion failure is represented by the CA size in µm^3 shown as mean ± SD with the number of embryos analysed in brackets. The significant increase in the CA size in *Vangl2*$^{+/Lp}$/*Daam1*$^{+/gt}$ and *Vangl2*$^{+/Lp}$/*Grhl3*$^{+/Ct}$ vs *Vangl2*$^{+/Lp}$ embryos was determined by unpaired *t* test (P=0.0002 and P<0.0001, respectively). Late NT closure progression is represented by the percentage of CAs associated to a closed NT, including the total number of CAs studied in brackets. Tail curvature, in mm^{-1}, is represented by the mean ± SD of the inverse of the radius of the best-fit circumference plotted along the curvature of the tail, with the number of embryos analysed in brackets. Since the tail grows over time, this parameter was measured exclusively in E12.5 embryos. The significant changes in tail curvature observed in *Vangl2*$^{+/Lp}$/*Grhl3*$^{+/Ct}$ embryos and *Vangl2*$^{+/Lp}$ embryos exposed to D-*chiro*-inositol vs NS *Vangl2*$^{+/Lp}$ embryos was determined by ordinary one-way ANOVA followed by Dunnett's multiple comparisons test (P=0.0041 and P=0.0164, respectively). * P<0.05; ** P<0.01; *** P<0.001; **** P<0.0001.

Table S2. Neural tube closure failure and CA characteristics along the anteroposterior axis.

Genotype	Condition	No. CAs per embryo			CA size (×10^5 µm^3)			CAs associated to closed NT (%)		
		≤29s	30-33s	≥34s	≤29s	30-33s	≥34s	≤29s	30-33s	≥34s
Embryos E11.5-13.5										
Vangl2$^{+/Lp}$	NS	0.8±1.1<br>(42)	1.3±0.8<br>(66)	0.2±0.4<br>(8)	0.94±1.39<br>(52)	1.60±1.52<br>(52)	0.20±0.65<br>(52)	33.3%<br>(14/42)	19.7%<br>(13/66)	0.0%<br>(0/8)
Embryos E12.5-13.5										
Vangl2$^{+/Lp}$	NS	0.7±1.1<br>(31)	1.5±0.8<br>(62)	0.2±0.5<br>(8)	0.82±1.40<br>(42)	1.79±1.53<br>(42)	0.25±0.72<br>(42)	45.2%<br>(14/31)	20.9%<br>(13/62)	0.0%<br>(0/8)
Vangl2$^{+/Lp}$/Daam1$^{+/gt}$	NS	0.7±0.9<br>(8)	1.5±0.7<br>(18)	0.1±0.3<br>(1)	1.41±2.85<br>(12)	**4.22±3.33**********<br>(12)	0.15±0.52<br>(12)	37.5%<br>(3/8)	27.8%<br>(5/18)	100%<br>(1/1)
Embryos E11.5-12.5										
Vangl2$^{+/Lp}$	NS	0.8±0.8<br>(33)	1.2±0.8<br>(50)	0.2±0.5<br>(8)	0.96±1.20<br>(42)	1.58±1.28<br>(42)	0.25±0.72<br>(42)	24.2%<br>(8/33)	8.0%<br>(4/50)	0.0%<br>(0/8)
Vangl2$^{+/Lp}$/Grhl3$^{+/Ct}$	NS	**2.4±1.5**********<br>(29)	1.5±0.8<br>(18)	0.3±0.9<br>(4)	**7.75±4.87**********<br>(12)	2.85±1.82<br>(12)	0.26±0.60<br>(12)	13.8%<br>(4/29)	27.7%<br>(5/18)	25.0%<br>(1/4)
Embryos E12.5										
Vangl2$^{+/Lp}$	NS	0.7±0.8<br>(22)	1.4±0.7<br>(46)	0.3±0.5<br>(8)	0.81±1.15<br>(32)	1.82±1.21<br>(32)	0.33±0.81<br>(32)	36.4%<br>(8/22)	8.7%<br>(4/46)	0%<br>(0/8)
Vangl2$^{+/Lp}$	FA	0.9±0.7<br>(14)	1.6±0.9<br>(24)	0.1±0.4<br>(2)	1.33±1.42<br>(15)	2.00±1.47<br>(15)	0.42±1.32<br>(15)	28.6%<br>(4/14)	16.7%<br>(4/24)	0%<br>(0/2)
Vangl2$^{+/Lp}$	MI	1.1±0.5<br>(12)	1.2±0.9<br>(13)	0.3±0.5<br>(3)	1.54±1.61<br>(11)	1.47±1.49<br>(11)	0.19±0.41<br>(11)	8.3%<br>(1/12)	7.7%<br>(1/13)	0%<br>(0/3)
Vangl2$^{+/Lp}$	CI	1.2±1.0<br>(21)	1.6±0.7<br>(27)	0.2±0.5<br>(3)	1.30±1.21<br>(17)	1.73±1.33<br>(17)	0.18±0.51<br>(17)	28.6%<br>(6/21)	0%<br>(0/27)	0%<br>(0/3)
Vangl2$^{+/Lp}$	FA+CI	1.2±1.0<br>(13)	1.5±0.7<br>(16)	0.2±0.4<br>(2)	1.43±1.54<br>(11)	1.93±1.27<br>(11)	0.36±0.94<br>(11)	30.8%<br>(4/13)	6.2%<br>(1/16)	0%<br>(0/2)

This table represents the anteroposterior distribution of dorsal fusion failure ('Number of CAs in each zone per embryo' and 'CA size') as well as the late neural tube closure ('CAs associated to closed neural tube') in the different experimental groups of the study: *Vangl2$^{+/Lp}$* embryos; the double mutants *Vangl2$^{+/Lp}$/Daam1$^{+/gt}$* and *Vangl2$^{+/Lp}$/Grhl3$^{+/Ct}$*, and *Vangl2$^{+/Lp}$* embryos under different supplementation conditions: NS, not supplemented; FA, supplemented with folic acid; MI, supplemented with *myo*-inositol; CI, supplemented with D-*chiro*-inositol; FA+CI, supplemented with the combination of folic acid and D-*chiro*-inositol. The number of CAs in each zone the CA size (in µm^3) are shown as mean ± SD with the number of CAs and embryos, respectively, shown in brackets. The late NT closure progression is represented by the percentage of CAs associated to a closed NT, with the total number of CAs studied shown in brackets. The significant increase in the number and size of CAs in the ≤29s zone in *Vangl2$^{+/Lp}$/Grhl3$^{+/Ct}$* embryos and in the CA size in the 30-33s zone in *Vangl2$^{+/Lp}$/Daam1$^{+/gt}$* embryos compared to *Vangl2$^{+/Lp}$* was determined by two-way ANOVA followed by Šídák's multiple comparisons test. **** *P*<0.0001.

Table S3. Effects of different maternal supplementation conditions on the characteristics of litters and pups.

Condition	Dam weight gain (g)	Litter size	Live embryos (%)	Embryo genotype	Mendelian distribution (%)	Crown-rump length (mm)	Open spina bifida (%)	Exencephaly‡ (%)	Craniorachischisis (%)
**NS**	5.7±1.4 (14)	7.4±1.7 (14)	84.6 % (88/104)	***Vangl2*** $^{+/+}$	27.3% (24/88)	9.6±0.8 (23)	0%	0%	0%
				Vangl2 $^{+/Lp}$	50.0% (44/88)	9.1±0.9 (37)	0%	3.4% (3/88)	0%
				Vangl2 $^{Lp/Lp}$	22.7% (20/88)	8.8±0.7 (14)	0%	0%	100% (20/20)
**FA**	5.5±1.2 (9)	8.0±1.3 (9)	88.9% (64/72)	***Vangl2*** $^{+/+}$	31.3% (20/64)	9.4±0.8 (14)	0%	0%	0%
				Vangl2 $^{+/Lp}$	53.1% (34/64)	9.2±0.7 (29)	1.6% (1/64)	0%	0%
				Vangl2 $^{Lp/Lp}$	15.6% (10/64)	9.1±0.3 (10)	0%	0%	100% (10/10)
**MI**	5.2±1.3 (9)	6.1±1.2 (9)	74.5% (41/55)	***Vangl2*** $^{+/+}$	34.1% (14/41)	10.0±0.7 (13)	0%	0%	0%
				Vangl2 $^{+/Lp}$	36.6% (15/41)	9.5±1.2 (12)	2.4% (1/41)	0%	0%
				Vangl2 $^{Lp/Lp}$	29.3% (12/41)	9.6±0.8 (9)	0%	0%	100% (12/12)
**CI**	**4.4±1.1*** (16)	7.1±1.5 (16)	78.9% (90/114)	***Vangl2*** $^{+/+}$	17.8% (16/90)	9.8±0.3 (9)	0%	0%	0%
				Vangl2 $^{+/Lp}$	53.3% (48/90)	8.9±0.7 (39)	0%	2.2% (2/90)	0%
				Vangl2 $^{Lp/Lp}$	28.9% (26/90)	8.7±0.6 (17)	0%	0%	100% (26/26)
**FA+CI**	5.0±1.3 (9)	8.2±1.2 (9)	79.7% (59/74)	***Vangl2*** $^{+/+}$	37.3% (22/59)	9.3±0.5 (21)	0%	0%	0%
				Vangl2 $^{+/Lp}$	40.7% (24/59)	9.3±0.7 (24)	1.7% (1/59)	1.7% (1/59)	0%
				Vangl2 $^{Lp/Lp}$	22.0% (13/59)	8.7±0.4 (9)	0%	0%	100% (13/13)

This tables summarises the characteristics of litters and pups of *Vangl2* $^{+/Lp}$ dams under different supplementation conditions: NS, not supplemented; FA, supplemented with 10 ppm folic acid; MI, supplemented with 800 µg/g/day of *myo*-inositol; CI, supplemented with 800 µg/g/day D-*chiro*-inositol; FA+CI, supplemented with a combination of 10 ppm FA and 800 µg/g/day of CI. Dam weight gain (from E0.5 to E12.5), litter size, and crown-rump length are shown as mean ± SD, with the number of dams, litters, and embryos, respectively, shown in brackets. The percentage of live embryos was calculated by dividing the number of live embryos (subtracting the reabsorptions) by the total number of implants, shown in brackets. The Mendelian distribution and NTDs incidence (open spina bifida, exencephaly, and craniorachischisis) are shown as percentages with the number of embryos shown in brackets. The significant decrease in weight gain observed in *Vangl2* $^{+/Lp}$ dams supplemented with CI vs NS dams was determined by one-way ANOVA followed by Dunnett's multiple comparison test (P=0.0285). This test was also used to study the litter size, percentage of live embryos, and crown-rump length. The Mendelian distribution was studied by Chi-square test. * P<0.05.

‡We observed the exencephaly phenotype –which had not been previously associated with the Wnt-PCP pathway– in all supplementation conditions except *myo*-inositol. This phenotype appeared after confinement for the Covid-19 pandemic, when the mouse line was maintained with a reduced number of animals. This strategy, as recently published (Moncaut and Hart-Johnson, 2021), may have been the cause of the inevitable acceleration of genetic drift and accumulation of mutations. This would also explain why this phenotype was not seen after myo-inositol supplementation, as these experiments were done before confinement.

Table S4. List of antibodies used in this study.

**Primary Antibodies**	**Source**	**Dilution**	**Supplier**	**Reference**
β-Catenin	Mouse	1:150	BD Biosciences	610153
CD44	Mouse	1:100	BD Biosciences	554869
Caspase-3	Rabbit	1:500	Cell Signaling	9661S
E-Cadherin	Mouse	1:200	BD Biosciences	610181
Laminin	Rabbit	Undiluted	Abcam	b11575
N-Cadherin	Mouse	1:100	Invitrogen	18-0224
Pax3	Mouse	1:200	Hybridoma Bank	AB_528426
PDGFR-α	Rat	1:100	BD Biosciences	558774
Phospho-histone H3	Rabbit	1:500	Upstate Biotechnology	06-570
Slug	Mouse	1:100	Hybridoma Bank	62.1E6-S
Sox10	Guinea pig	1:500	Gift from M. Wegner, Erlangen, Germany	Stolt et al., 2003
ZO-1	Rabbit	1:150	Invitrogen	40-2200
**Secondary Antibodies**	**Source**	**Dilution**	**Supplier**	**Reference**
α-guinea pig (FITC)	Donkey	1:200	Jackson ImmunoResearch	706-095-148
α-mouse (Cy3)	Goat	1:300	Jackson ImmunoResearch	115-165-166
α-rabbit (Alexa Fluor 568)	Donkey	1:500	Invitrogen	A10042
α-rabbit (FITC)	Goat	1:250	Abcam	Ab6717
α-rat (Cy2)	Donkey	1:200	Jackson ImmunoResearch	712-225-153

Supplementary Materials and Methods

Protocol to assess the severity of the caudal neural tube fusion failure in *Vangl2*$^{+/Lp}$ embryos using the cellular aggregates (CAs) as a readout.

We have used whole mount *in situ* hybridisation for *Sox10* and corresponding sections to establish a protocol to assess the degree of severity of dorsal fusion impairment. The following parameters were measured.

1. ***Incidence.*** The parameter incidence refers to the percentage of embryos that present the phenotype, in this case the presence of cellular aggregates (CAs). This parameter can be measured from stereoscopic images of whole embryos after *in situ* hybridisation. Incidence can be considered as an indicator of the severity of dorsal neural tube (NT) closure failure. A high severity of the phenotype would correlate with a high incidence, as observed in *Vangl2*$^{+/Lp}$ embryos that present a 100% incidence, whereas a milder phenotype would imply a lower incidence.
2. ***Number of regions affected by NT fusion failure***. Embryos can present one or more CAs, reflecting the number of regions affected by NT fusion failure. The number of CAs per embryo can be assessed from stereoscopic images of whole embryos after *in situ* hybridisation for *Sox10*. For this parameter, 0 is considered when an embryo does not present any CAs in a given zone. A worsening of the phenotype would imply an increase in the number of CAs per embryo, and an improvement a reduction therein.
3. ***Location of NT fusion failure.*** The study of whole embryos revealed that most embryos developed more than one CA (Fig. 4B) and these were located within 3 specific zones: zone anterior to somite 29 (≤29s; Fig. 4D); intermediate zone, between somites 30 and 33 (30-33s; Fig. 4E,F); and the caudal zone, posterior to somite 34 (≥34s; Fig. 4G). The location of CAs with respect to these three defined anteroposterior regions can be assessed from stereoscopic images of whole embryos after *in situ* hybridisation for *Sox10*. As the presence of anterior CAs seems to be associated with an increase in the severity of the defect, worsening of the phenotype would lead to a greater presence of CAs in anterior positions, while a shift of the presence of CAs to more caudal areas would imply a milder phenotype.
4. ***NT affected by dorsal fusion failure***. Given that the CA appeared to fill the space left by a dorsal fusion failure, the size of the CAs can be measured as a readout of NT damage. To do so, CA area can be measured to from 40x micrographs of transverse sections (e.g., using ImageJ polygon tool or similar) noting that the area taken into consideration is the entire CA, as observed morphologically, and is not limited to the area marked with *Sox10*. The extent of damage in the dorsal NT is then calculated by summing up the areas of all the CAs found in an embryo, and multiplying this number by the thickness of the sections. For this parameter,

Disease Models & Mechanisms • Supplementary information

0 is considered when an embryo does not present any CAs in a given zone. For this parameter, the severity of the phenotype correlated directly with the size of CAs, and thus, an improvement of the phenotype would result in a reduction of CA size, and a worsening of the phenotype would imply larger CAs.

5. ***Late NT closure progression*.** In what we have termed "late NT closure progression" we found that some CAs were below/over a closed NT, indicating that although CAs formed where the NT had failed to fuse, the presence of the CA may have facilitated late closure, thereby attenuating dorsal damage. Thus, an increase in CAs associated with a closed NT would be related to an improvement of the phenotype, while an increase in CAs associated with an open NT would be expected to be found with more severe phenotypes. This parameter can be evaluated by visually determining whether each CA is associated with a closed or open NT using 40x micrographs of transverse sections.
6. ***Tail curvature***. One of the most obvious characteristics of *Vangl2*$^{+/Lp}$ mutants is the presence of a curled tail. The curvature of the caudal region or tail curvature (κ) can be measured from stereoscopic images of whole embryos by measuring the radius (r) of a circumference that is drawn (e.g., using ImageJ software) following the curvature of the tail embryos and applying the formula $\kappa=1/r$. Since this parameter is associated with caudal damage (De Castro et al., 2018), it would be expected that a greater curvature of the tail turn would be associated with more severe phenotypes than a lesser curvature.

REFERENCES

De Castro, S. C. P., Gustavsson, P., Marshall, A. R., Gordon, W. M., Galea, G., Nikolopoulou, E., Savery, D., Rolo, A., Stanier, P., Andersen, B. et al. (2018). Overexpression of Grainyhead-like 3 causes spina bifida and interacts genetically with mutant alleles of Grhl2 and Vangl2 in mice. *Hum Mol Genet* **27**, 4218-4230.

Moncaut, N. and Hart-Johnson, S. (2021). The impact of COVID-19 lockdowns on the genetic integrity of your mouse colonies. *Lab Anim (NY)* **50**, 301-302.
